# Transcriptional changes are tightly coupled to chromatin reorganization during cellular aging

**DOI:** 10.1111/acel.14056

**Published:** 2023-12-07

**Authors:** Jana M. Braunger, Louis V. Cammarata, Trinadha Rao Sornapudi, Caroline Uhler, G. V. Shivashankar

**Affiliations:** ^1^ Eric and Wendy Schmidt Center Broad Institute of MIT and Harvard Cambridge Massachusetts USA; ^2^ Department of Statistics Harvard University Cambridge Massachusetts USA; ^3^ Division of Biology and Chemistry Paul Scherrer Institute Villigen Switzerland; ^4^ Laboratory for Information and Decision Systems Massachusetts Institute of Technology Cambridge Massachusetts USA; ^5^ Department of Health Sciences and Technology ETH Zurich Zurich Switzerland

**Keywords:** 3D genome organization, cellular aging, gene expression, network analysis, transcription factors

## Abstract

Human life expectancy is constantly increasing and aging has become a major risk factor for many diseases, although the underlying gene regulatory mechanisms are still unclear. Using transcriptomic and chromosomal conformation capture (Hi‐C) data from human skin fibroblasts from individuals across different age groups, we identified a tight coupling between the changes in co‐regulation and co‐localization of genes. We obtained transcription factors, cofactors, and chromatin regulators that could drive the cellular aging process by developing a time‐course prize‐collecting Steiner tree algorithm. In particular, by combining RNA‐Seq data from different age groups and protein–protein interaction data we determined the key transcription regulators and gene regulatory changes at different life stage transitions. We then mapped these transcription regulators to the 3D reorganization of chromatin in young and old skin fibroblasts. Collectively, we identified key transcription regulators whose target genes are spatially rearranged and correlate with changes in their expression, thereby providing potential targets for reverting cellular aging.

## Graphical abstract

We present a prize‐collecting Steiner tree algorithm to discover key transcription regulators in cellular aging from time‐course RNA‐Seq data. Integrating Hi‐C data, our analysis demonstrates spatial rearrangements among the target genes controlled by these regulators during aging. Our findings suggest a tight coupling between changes in co‐regulation and co‐localization of age‐associated genes, thereby providing potential targets for reverting cellular aging.

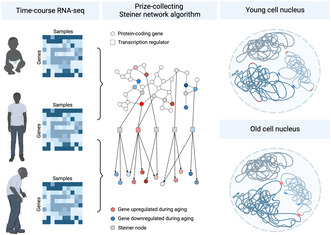

**Abbreviations**
DE: differentially expressed
ECM: extracellular matrix
FDR: false discovery rate
FPKM: fragments per kilobase of transcript per million
FWER: family‐wise error rate
GO: gene ontology
Hi‐C: high throughput chromosome conformation capture
LAS: large average submatrix
PPI: protein‐protein interaction
TAD: topologically associated domain
TF: transcription factor

## 1 INTRODUCTION

Aging is characterized by a gradual decline of physiological integrity, resulting in compromised function and increased vulnerability to disease and mortality (López‐Otín et al., 2023). At the molecular level, this process involves systematic changes in gene expression programs and pathways, accompanied by alterations in cellular organization and function at various scales, including cytoskeletal architecture and chromatin structure (Zhang, Qu, et al., 2020). Characterizing the key transcriptional drivers of aging is crucial to advance our understanding of this process and develop therapies to mitigate its effects. This is challenging due to the complex and distinct impacts of aging on gene expression and molecular pathways across different cell types and tissues. Recent evidence, such as a meta‐analysis of transcriptomic aging signatures across multiple tissues, has shown limited overlap between organs (Palmer et al., 2021). Nevertheless, several studies have found common patterns of gene expression changes associated with aging across various tissues (e.g., genes involved in inflammation and immune response, cell cycle, collagen processing, and metabolism and mitochondrial functions) (de Magalhães et al., 2009; Palmer et al., 2021).

While multiple studies have examined age‐associated changes in gene expression and proposed several common as well as cell type‐specific gene signatures of aging (Lee & Shivashankar, 2020; Stegeman & Weake, 2017), a systematic description of the corresponding transcriptional regulatory programs has yet to be established. Given that transcription factors, cofactors, and chromatin regulators (which we collectively refer to as transcription regulators or “TFs”) play a pivotal role in determining cell identity and function, it is particularly critical to investigate how their regulatory interactions are disrupted or changed during aging (Maity et al., 2022). Assessing the role of TFs in aging based solely on transcriptomic data presents significant challenges. First, the impact of TFs may not necessarily correlate with age‐associated changes in the expression of the genes that code for these TFs. For example, while some TFs may exhibit minimal or inconsistent expression changes during aging, their subcellular localization and the accessibility of their target genes can influence their regulatory significance in a time‐dependent manner. Second, for TFs that display consistent expression changes throughout aging, the effect size of these changes tend to be small due to increased stochasticity in gene expression associated with aging (Levy et al., 2020; Martinez‐Jimenez et al., 2017). To address these challenges, a recent study leveraged the expression scores of TF regulons to infer TF activity (Maity et al., 2022). This approach has a higher signal‐to‐noise ratio for identifying an age‐associated TF compared to using the TF's expression alone but suffers from two main limitations. First, regulon databases can be incomplete and thus important regulatory activity can be missed. Second, while such an approach globally identifies age‐associated TFs, it does not provide a time‐dependent assessment of their significance.

Recently, the time‐dependent regulatory control of transcriptional programs has been linked to the 3D structure of the genome thanks to advances in chromosomal conformation capture methods (Bickmore & van Steensel, 2013; Dekker & Mirny, 2016; Lieberman‐Aiden et al., 2009; Schmitt et al., 2016). Inside the cell nucleus, chromosomes are organized in a nonrandom fashion, such that each chromosome occupies its own territory. At the intrachromosomal level, DNA sequences may interact in the form of topologically associated domains (TADs) (Acemel & Lupiáñez, 2023; Dixon et al., 2012). In addition, regions on neighboring chromosomes may loop out and intermingle with each other (Maass et al., 2019). Several studies have suggested that these interchromosomal regions could harbor co‐regulated gene clusters (Bashkirova & Lomvardas, 2019; Belyaeva et al., 2017). Transcription factories (or hubs or condensates), corresponding to the clustering of genes, transcriptional machinery, and regulatory factors, have been proposed as a model for gene regulation (Belyaeva et al., 2017; Chen et al., 2015; Palacio & Taatjes, 2022; Papantonis & Cook, 2013; Uhler & Shivashankar, 2017). As tissues age, the extracellular matrix (ECM) stiffness and cell‐ECM interactions are altered, leading to transformations in cell shape and behavior (Uhler & Shivashankar, 2020). This affects the type of gene programs expressed by the cell through changes in 3D chromatin organization and the subcellular compartmentalization of key TFs (Mitra et al., 2017). Yet, to date, little progress has been made in connecting the transcriptional space and the 3D chromatin packing landscape in the context of aging.

In this work, we integrate time‐course transcriptomic data, TF ChIP‐Seq data, and protein–protein interaction (PPI) data to characterize the evolving transcriptional regulatory programs during aging in human skin fibroblasts. We construct gene signatures of skin fibroblasts at different time points, and we identify key TFs that could explain the chronological progression through these gene signatures using a network‐based approach. The incorporation of PPI data helps alleviate potential issues stemming from incomplete regulatory relationships. Moreover, our approach provides a time‐dependent assessment of the roles played by TFs throughout the aging process. Furthermore, we connect our findings on aging transcriptional regulatory programs to the 3D chromatin packing landscape using chromosomal conformation capture data of young and old human skin fibroblasts. We find that the signature genes of aging regulated by age‐associated TFs tend to spatially co‐cluster in a time‐dependent manner. Collectively, our findings shed new light on the tight coupling between transcriptional changes and chromatin reorganization during cellular aging and also identify key TFs that exhibit time‐dependent regulatory patterns during cellular aging. These TFs may hold promise as potential therapeutic targets to mitigate physiological decline and reduce the occurrence of age‐related disease.

## 2 RESULTS

### 2.1 Differential gene expression analysis identifies genes with age‐dependent expression

During aging, many changes occur in the human transcriptome, and there are also differences in the aging gene expression signatures between tissues and cell types. Characterizing these aging gene signatures is an important step toward potential therapies to delay the onset of age‐associated cellular decline (Stegeman & Weake, 2017). To identify genes that are differentially expressed during the human lifespan in a tissue‐specific manner, we used bulk RNA‐Seq data measuring the gene expression of skin fibroblasts from individuals between 1 and 96 years (Fleischer et al., 2018). In total, the dataset includes 133 individuals, out of which 74% are male; see Figure S1. Given the highly significant correlation between female and male transcriptomic profiles throughout aging (Figure S1), we did not perform additional gender‐specific analyses.

**FIGURE 1:**
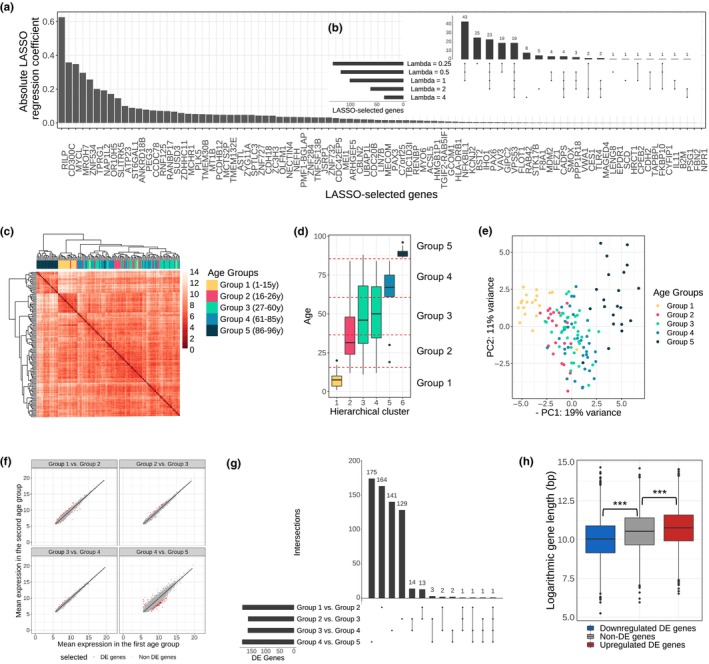
Differential gene expression analysis identifies age‐associated gene signatures that vary across age groups. (a) Absolute coefficients resulting from the LASSO regression of age on gene expression using the RNA‐Seq data from all individuals (penalty *λ* = 1). (b) Intersection of genes with nonzero LASSO coefficient for varying penalties (*λ* ∈ {0.25, 0.5, 1, 2, 4}). (c) Hierarchical clustering (Euclidean metric, ward.D2 linkage) of all 133 individuals in the dataset based on the variance‐stabilized transformed expression of genes selected in A. (d) Age distribution in clusters of individuals obtained by thresholding the dendrogram in C (six clusters using a phenetic threshold = 17). We defined five age groups as indicated by the red horizontal dashed lines. Each box is colored according to the age group it contributes most to. As Clusters 3 and 4 cover the same range, they have the same color. (e) PCA plot of individuals using the variance‐stabilized transformed RNA‐Seq counts of genes selected in A. Each individual is colored according to its age group. (f) Mean variance‐stabilized RNA‐Seq counts between consecutive age groups. DE genes (see thresholds in Figure S2C) are marked in red for each of the four transitions between age groups. (g) DE genes between pairs of consecutive age groups. Thresholds in Figure S2C were used to identify around 170 DE genes for each transition and the overlap of these groups of DE genes in the four transitions between age groups is shown. (h) Age‐associated gene length imbalance analysis. The boxplot shows the logarithmic gene length for downregulated DE genes (blue), non‐DE genes (gray), and upregulated DE genes (red) between Group 1 and Group 5.

First, we sought to identify a set of key genes for the aging process. Because gene signatures of aging are known to be tissue and cell type‐specific (Stegeman & Weake, 2017), we inferred aging‐associated genes using the bulk RNA‐Seq data from human skin fibroblasts. For gene selection we used linear regression with age as the predicted variable and RNA counts as predictors with a LASSO penalty. We report the genes with nonzero regression coefficients in Figure 1a. Selecting a penalty parameter of *λ* = 1 provided robust results (Figure 1b) resulting in 101 genes with age‐associated expression.

Based on this set of age‐related genes, a hierarchical clustering approach was performed to define five age groups (see Figure 1c,d and Methods for details): 1–15 years (Group 1, 19 individuals), 16–26 years (Group 2, 20 individuals), 27–60 years (Group 3, 39 individuals), 61–85 years (Group 4, 34 individuals), and 86–96 years (Group 5, 21 individuals). While the youngest and oldest individuals are clearly separated from individuals in the other age groups, the clusters containing individuals of middle ages are more mixed, as shown in the PCA plot of Figure 1e.

Next, we studied the transitions from one age group to the next. There are four such transitions: Group 1 to Group 2, Group 2 to Group 3, Group 3 to Group 4, and Group 4 to Group 5. For each transition, approximately 170 differentially expressed (DE) genes were selected using FDR‐adjusted *p*‐value based on the fold change and robustness of a gene using subsampling of the individuals in each age group (Figure S2A–C and Methods). Overall, the difference in gene expression was much higher in the last transition (between Group 4 and 5); see Figure 1f. This suggests that more genes change their expression from Group 4 to Group 5 (the oldest age group with individuals above 85 years). This observation is in accordance with a previous study (Márquez et al., 2020) which identified more rapid transcriptional and epigenomic changes with high age instead of gradual linear changes with age. To select approximately the same number of DE genes as in the other transitions, for the subsequent analysis we used a lower *p*‐value threshold for the last transition (Figure S2C).

Interestingly, by analyzing the DE genes selected in each of the four age group transitions we found that most of the DE genes were only DE in one of the four transitions (Figure 1g). This suggests that aging gradually turns on different gene programs. GO analysis of all identified DE genes revealed that they were significantly involved in programs including extracellular matrix organization, immune response, cellular proliferation, as well as cell cycle processes, which all play important roles during aging (Figure S2D) (López‐Otín et al., 2023). Even though most of the DE genes were selected only for one transition, many of them not only changed their expression during this transition but also experienced consistent changes (although not significant) in their expression throughout all five age groups (Figure S3).

A recent study of the aging transcriptome revealed an age‐related transcript length imbalance (Stoeger et al., 2022). To connect our results to this study, we examined the relationship between gene length and expression between young and old individuals in the skin fibroblast dataset. DESeq2 was used with an adjusted *p*‐value threshold of 0.05 to obtain the genes that are differentially expressed between Group 1 (youngest individuals) and Group 5 (oldest individuals). Genes that were significantly downregulated between the youngest (Group 1) and the oldest (Group 5) age group had significantly shorter transcripts than non‐DE genes (Figure 1h, *p*‐value <2.2e‐16, one‐sided Welch *t* test). Furthermore, genes that were upregulated during aging (positive fold change between Group 1 and Group 5) were significantly longer (*p*‐value = 1e‐6, one‐sided Welch *t* test). This is consistent with the previously observed positive correlation of transcript length and fold change of a gene in human skin fibroblasts (Stoeger et al., 2022).

### 2.2 Steiner tree analysis reveals key aging‐associated transcription regulators

Transcription factors, cofactors, and chromatin regulators are critical for regulating the expression of gene programs involved in aging. To identify TFs that could explain the evolution from the DE genes in one age transition to the DE genes in the next age transition, we developed a network‐based approach using prize‐collecting Steiner trees (Huang & Fraenkel, 2009; L. Yuan et al., 2022). At a high level, we used a PPI network augmented with regulatory edges between TFs and their target genes, where the nodes were prized based on differential expression and edge costs were assigned based on edge confidence (see SI Methods). The prize‐collecting Steiner tree algorithm was used to identify the most efficient way to connect the DE genes in one transition to the DE genes in the next transition. This approach identifies key TFs and other intermediary genes that are needed to connect these DE genes and could therefore play an important role in driving the aging process.

More precisely, to study the evolution from the DE genes between Group 1 and Group 2 (which we refer to as the source DE genes) to the DE genes between Group 2 and Group 3 (referred to as the target DE genes), we built a gene network in the following way. First, we retrieved human PPI data from the STRING database (Szklarczyk et al., 2019) (17,954 proteins and 1,900,568 physical interactions between pairs of proteins) and we filtered out proteins corresponding to inactive genes in both age groups (Group 1 and Group 2). Gene activity was defined by thresholding the RNA‐Seq expression distribution as seen in Figure S5A. In the resulting gene network, we prized the source DE genes with their absolute log2‐fold change between Group 1 and Group 2. Second, nodes were added separately for the target DE genes, and they were prized with their absolute log2‐fold change between Group 2 and Group 3. To connect the gene network with the target DE gene nodes, we added edges between TFs in the gene network to their regulatory targets in the target DE genes using TF‐target regulatory data from hTFtarget (Zhang, Liu, et al., 2020), which is a database containing 1,319,123 regulatory links between 495 TFs and 38,183 targets (see Methods). The resulting network is referred to as network N1. We also created networks to study the evolution from the DE genes between Group 2 and Group 3 to the DE genes between Group 3 and Group 4 (network N2) and the evolution from the DE genes between Group 3 and Group 4 to the DE genes between Group 4 and Group 5 (network N3).

**FIGURE 2:**
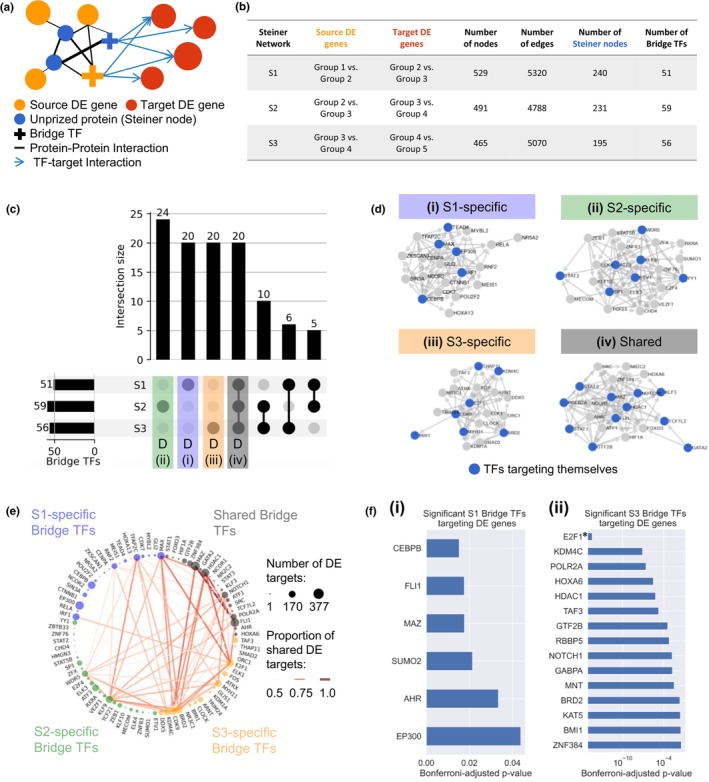
Steiner tree analysis reveals key transcription factors that could drive the aging process. (a) Schematic of our prize‐collecting Steiner tree methodology to identify key transcription factors (TFs) that connect DE genes in one age group transition (source DE genes, e.g., Group 1 vs. Group 2) to DE genes in the next age group transition (target DE genes, e.g., Group 2 vs. Group 3). An initial network was created by adding a set of regulatory edges (blue arrows) to the human protein–protein interaction (PPI) network (black edges, thickness represents edge cost, which is inversely proportional to the confidence of an edge in the PPI network). The blue regulatory edges connect TFs to their target genes that are part of the target DE genes. Node prizes (represented by node size) were added for the source DE genes (orange nodes) and the target DE genes (red nodes) according to their log2 fold change. The prize‐collecting Steiner tree algorithm was used to identify a subnetwork, the Steiner network, that connects the orange prized nodes to the red prized nodes by minimizing the overall edge cost; see Methods. This identifies a set of bridge TF genes (crosses) as well as other unprized genes (Steiner nodes, in blue) that are required to connect the source and target DE genes. (b) Descriptive statistics of the three Steiner networks constructed as described in A: For each of the networks S1, S2, and S3 different age groups were used to define the source and target DE genes. The number of nodes, edges, Steiner nodes and bridge TFs in the identified Steiner networks are shown. (c) UpSet plot of bridge TFs selected in the three Steiner networks (S1, S2, and S3). The first four groups (S2‐specific bridge TFs in green, S1‐specific bridge TFs in purple, S3‐specific bridge TFs in yellow, and TFs occurring in all three Steiner networks in grey) are colored to match them with the groups used in D. (d) Networks of regulatory relationships (retrieved from hTFtarget) among bridge TFs that are specific to Steiner network S1 (d(i), purple), Steiner network S2 (d(ii), green), Steiner network S3 (d(iii), yellow), and bridge TFs that were shared across all three networks (d(iv), grey). TFs targeting themselves are shown in blue. In the network of shared bridge TFs, the number of edges per node is significantly higher than in networks obtained by selecting 20 random TFs among the ones included in at least one Steiner network (1000 simulations with random bridge TFs, *p*‐value = 0.047). (e) Co‐target network among the 4 bridge TF groups in D: S1‐specific bridge TFs (blue nodes), S2‐specific bridge TFs (green nodes), S3‐specific bridge TFs (orange nodes), and shared bridge TFs (black nodes). Two TFs are linked by an edge if they share significantly more DE targets than expected under a hypergeometric null model (*p* < 1e‐15) and if the proportion of the number of targets in the intersection divided by the union is bigger than 0.5. Edges are colored and sized by this proportion of shared DE targets and nodes are sized according to the number of DE targets each bridge TF has. (f) Adjusted *p*‐values for bridge TFs in Steiner network S1 (f(i)) and S3 (f(ii)) indicating their propensity to regulate target DE genes (red nodes in A). A small *p*‐value means that a TF targets more target DE genes in its corresponding network than expected by chance based on a hypergeometric null model using its number of targets in the whole genome as a baseline. The *p*‐values were Bonferroni‐adjusted and only TFs with an adjusted *p*‐value below 0.05 are reported. TFs with differential gene expression are marked by *.

Next, the prize‐collecting Steiner tree algorithm was used on each gene network (N1, N2, N3) to identify smaller subnetworks (S1, S2, S3), called Steiner networks, that connect the source and target DE genes while minimizing the overall cost of the included edges (Figure 2a, Figure S6, Methods). The resulting Steiner networks contain around 500 nodes and 5000 edges (Figure 2b). To visualize one example, the Steiner network corresponding to S3 is shown in Figure S6. In addition to the prized nodes, these Steiner networks also contain around 200 unprized nodes which are required to connect the prized nodes. Gene ontology analysis revealed that these unprized nodes (called Steiner nodes) in the three subnetworks are involved in aging relevant pathways such as cell proliferation or DNA repair (Figure S8).

Each of the three Steiner networks contains approximately 50–60 TFs that the algorithm selected to connect the source DE genes to the target DE genes. These *bridge TFs* help bridge DE genes in one age transition to DE genes in the subsequent age transition. The majority of identified bridge TFs were either specific to only one of the networks (S1‐specific, S2‐specific, or S3‐specific) or occurred in all three of them (shared) (Figure 2c). We created networks showing the regulatory relationships among the S1‐specific, S2‐specific, S3‐specific, and shared bridge TFs to assess how much the bridge TFs in each group target each other; this could function as a positive feedback loop for signal amplification. The identified bridge TF groups show dense regulatory relationships (Figure 2d). For example, the regulatory network of the shared bridge TFs includes many TFs that target themselves (blue nodes), as well as has a significantly higher network density than networks of random bridge TFs (*p*‐value = 0.047, permutation test). Interestingly, 37.1% of all identified bridge TFs target themselves, while this number is only 19.6% for all TFs in hTFtarget. This suggests a high level of reinforcement in the regulation of age‐dependent gene programs. Furthermore, the bridge TFs in the four groups (S1‐specific, S2‐specific, S3‐specific and shared across all three networks) also share many DE genes as their targets, that is, many pairs of bridge TFs share significantly more targets than expected under a hypergeometric null model (128 TF pairs with *p* < 1e‐15); see Figure 2e. Such overlap could be beneficial to add robustness to the transcriptional regulation during aging.

While some TFs are very specific and only target a few genes in the genome, other TFs target and regulate the expression of thousands of genes (Figure S4B). As TFs with many targets will also target many of the DE genes in our networks, it is likely for them to be included as bridge TFs in the Steiner networks. Using a hypergeometric null model and correcting for the Family‐Wise Error Rate (FWER, with a significance threshold at 0.05), we identified 6 bridge TFs in Steiner network S1 and 15 bridge TFs in S3 that are highly specific to their network and target significantly more target DE genes than genes overall (Figure 2f, Figure S9). These bridge TFs contain known key drivers of aging. For example, in S3, the bridge TF with the smallest *p*‐value is E2F1, which plays a central role in cell proliferation, development and apoptosis and regulates cellular senescence, one of the hallmarks of aging (Xie et al., 2014). E2F1 also shows strong expression changes throughout the different age groups (Figure S10).

### 2.3 Hi‐C analysis shows differences in interchromosomal intermingling during aging

It has been shown in previous studies that the spatial co‐localization of genes regulated by the same transcription regulators may facilitate their coordinated expression: Active clusters of genes that share TF binding sites and are enriched for transcriptional machinery are also known as transcription factories (Palacio & Taatjes, 2022). For example, target genes of the transcription factor KLF1 preferentially co‐localize in specialized transcription factories in murine erythroid tissues (Schoenfelder et al., 2010), and NFκB target genes in primary human endothelial cells congregate into discrete NFκB factories upon stimulation by TNFα (Papantonis et al., 2012). Several comprehensive data‐driven studies have confirmed that genes regulated by common transcription factors and genes that belong to the same functional groups that reside on distinct chromosomes have an enhanced tendency to be in spatial proximity (Belyaeva et al., 2017; Thévenin et al., 2014). As it is known that major changes in the three‐dimensional arrangement of the DNA in the nucleus occur during aging (O'Sullivan & Karlseder, 2012; Sun et al., 2018), we analyzed the chromatin organization changes in relation to the transcriptomic changes.

To study the chromatin reorganization during aging, we used Hi‐C data from our lab (GEO accession GSE237271) consisting of two replicates for young fibroblasts originating from a 10‐year‐old donor (GM09503), as well as two replicates for old fibroblasts from a 75‐year‐old donor (GM08401). A resolution of 250 kilo base pairs (kb) was chosen as a trade‐off between low and high resolution. In low resolutions, the bins are large and therefore contain many genes such that it is difficult to locate genes of interest. In high resolutions with smaller bins the detected Hi‐C contacts get sparser and noisier, which makes clustering more challenging, especially for interchromosomal contacts. With a resolution of 250 kb the genome is binned into 11,537 loci with up to 1000 loci for large chromosomes like chromosomes 1 or 2 and around 200 loci for the small chromosomes like chromosomes 21 or 22 (Figure S14A). For illustration, Figure S11 contains examples of intrachromosomal and interchromosomal Hi‐C contact maps at several resolutions. Global characterization of the Hi‐C contact maps in *cis* using distance decay plots (Figure S12) and insulation score profiles (Figure S13) did not reveal substantial differences between the young and old cell states at the intrachromosomal level.

**FIGURE 3:**
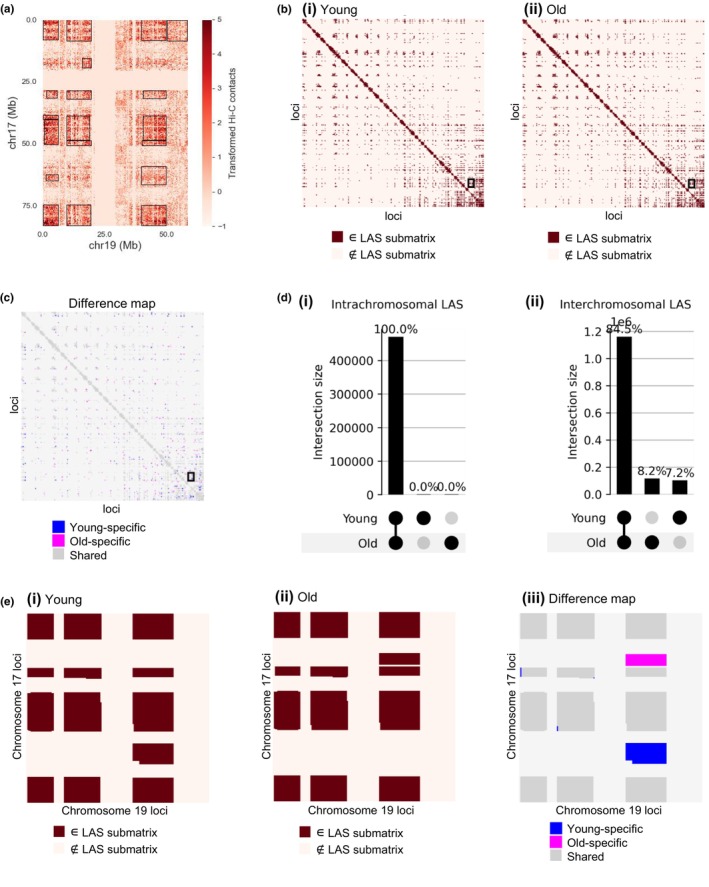
Hi‐C analysis shows differences in interchromosomal intermingling during aging. (a) Interchromosomal Hi‐C matrix of chromosome 17 and chromosome 19 for the young (replicate 1) sample. Hi‐C contact values were preprocessed and centerdized, see Methods. Black boxes indicate significant submatrices with high average values selected by the large average submatrix (LAS) algorithm. (b) Binarized intermingling map for young (b(i)) and old (b(ii)) human skin fibroblasts based on the identified LAS matrices in the Hi‐C data. All 250 kb loci are ordered according to their ascending genomic location (x‐axis from left to right and y‐axis from top to bottom) and all submatrices (dark red) identified with the LAS algorithm that were above the threshold in both replicates are shown. Interactions between chromosomes 17 and 19, for which a zoom‐in is shown in E, are marked with a black box. (c) Intermingling difference map as a comparison of the two binarized maps in B. It shows which of the LAS submatrices only occurred in young samples (blue), only in old samples (magenta) or in both samples (dark grey). Interactions between chromosomes 17 and 19 are enclosed in a black box. (d) UpSet plot of intrachromosomal LAS submatrices (d(i)) and interchromosomal LAS submatrices (d(ii)) selected in the young and old Hi‐C data, showing that the 3D chromatin rearrangements during aging are mainly interchromosomal. Intersection sizes are measured by the number of pixels in each submatrix. (e) Zoom‐in for interchromosomal contacts of chromosome 17 (y‐axis) with chromosome 19 (x‐axis). Based on the results of the LAS algorithm, the binarized intermingling maps for young (e(i)) and old (e(ii)) were created. On the right (e(iii)), the difference between those two maps is visualized by coloring submatrices only found in young samples in blue, only found in old samples in magenta, and the submatrices found in both young and old samples in grey.

To identify regions of chromosomal interactions, we applied the Large Average Submatrix (LAS) algorithm (Shabalin et al., 2009) to each intra‐ and interchromosomal Hi‐C contact matrix in each of the four Hi‐C datasets (two replicates in the young and old condition). This algorithm detects contiguous submatrices whose score (considering the size of the submatrix and the average value of its entries) is high compared to similar‐sized submatrices in the Hi‐C map (Figure 3a and SI Methods). Overall, we obtained around 3000 LAS submatrices per sample. The interchromosomal LAS scores of the two replicates in each condition were very similar with a correlation coefficient of 0.85 (*p*‐value <2e‐308, correlation test, see Methods) in the young condition and 0.94 (*p*‐value <2e‐308, correlation test, see Methods) in the old condition (Figure S14C). Therefore, the results for the two replicates per condition were combined, resulting in a set of LAS submatrices specific to the young cell state and a set of LAS submatrices specific to the old cell state. The binarized Hi‐C maps showing the identified LAS submatrices (i.e., contacts) among all loci for the young and old cell states are shown in Figure 3b.

To study the cell‐state specific intermingling, we computed the difference between the binarized Hi‐C maps for young and old fibroblasts. The resulting intermingling difference map among all loci is shown in Figure 3c. Interestingly, almost all intrachromosomal LAS submatrices were shared among young and old fibroblasts (Figure 3d(i), overlap of 99.995%), while 15.5% of the interchromosomal intermingling regions were specific to either the old cell state or the young cell state (Figure 3d(ii)). The rearrangements between the young and old states at the chromosome level are shown in Figure S14D and examples of difference maps are provided for chromosome 17 in *cis* (Figure S11A) and for the chromosome pair 17‐19 in *trans* (Figure 3e and Figure S11B). In the latter, one LAS submatrix was present only in the young condition (colored in blue) and one submatrix only in the old condition (colored in magenta). These results suggest that the intrachromosomal chromatin organization is rather stable during aging, and the chromatin reorganization is mostly happening via changes in interchromosomal contacts. Although interchromosomal interactions have been less studied in the past compared to intrachromosomal chromatin interactions, there has been increasing evidence supporting their role in cell type‐/state‐specific gene regulation (Maass et al., 2019).

### 2.4 Hi‐C analysis shows intermingling differences for differentially expressed genes

**FIGURE 4:**
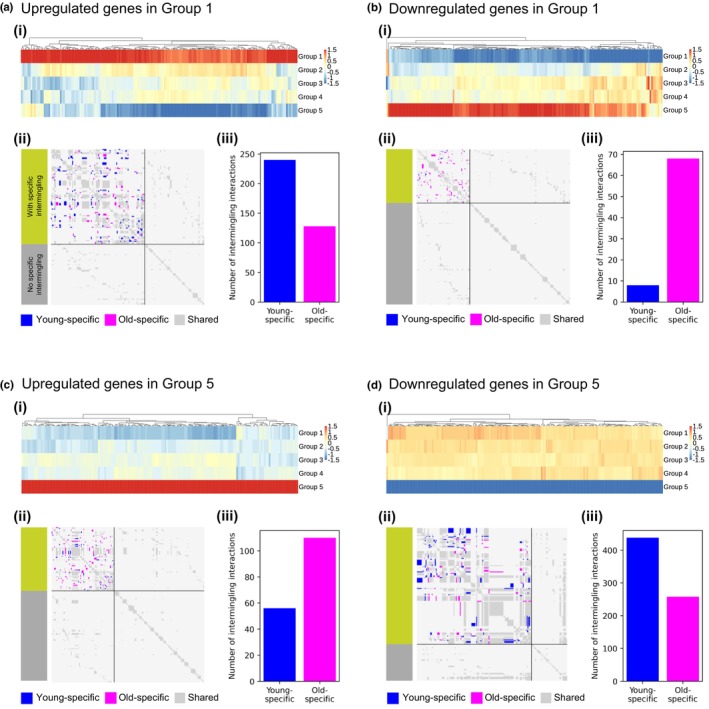
Hi‐C analysis shows intermingling differences for differentially expressed genes. (a) Expression and spatial clustering of upregulated genes in Group 1. a(i) shows the heatmap of the *z*‐scored expression of the genes upregulated in Group 1 over the five age groups from Group 1 (youngest, top) to Group 5 (oldest, bottom). Genes were ordered by hierarchical clustering with Euclidean distance and complete linkage. a(ii) shows the intermingling difference map for the genes in a(i). Hi‐C entries corresponding to the interaction of two DE genes that were only part of an LAS submatrix in young Hi‐C data are shown in blue, those only found in old Hi‐C data in magenta, and those that are in LAS submatrices occurring in young and old Hi‐C data in grey. The DE genes were grouped into the ones with specific intermingling (green group with blue and magenta spots) and the ones without specific intermingling (grey group). The DE genes per group were sorted according to their ascending genomic location. a(iii) shows a barplot quantifying the amount of young‐ and old‐specific intermingling entries in the intermingling difference maps. Here, 8.5% out of all intermingling interactions were young‐specific and 5.1% old‐specific. (b) Expression and spatial organization of downregulated genes in Group 1. In b(ii) and b(iii), 0.77% out of all intermingling interactions were young‐specific and 7.7% old‐specific. (c) Expression and spatial clustering of upregulated genes in Group 5. In c(ii) and c(iii), 3.5% out of all intermingling interactions were young‐specific and 6.7% old‐specific. (d) Expression and spatial organization of downregulated genes in Group 5. In d(ii) and d(iii), 6.4% out of all intermingling interactions were young‐specific and 4% old‐specific.

Leveraging the LAS clusters that we identified, we proceeded to investigate the intermingling of differentially expressed genes during aging. For this analysis, we obtained signature genes of the young state by performing a differential gene expression analysis of Group 1 (1–15 years) versus all others and selecting a similar number of genes as in the previously analyzed transitions (170 genes with smallest *p*‐values, Wald test, FDR‐adjustment) and similarly for Group 5 (86–96 years old) to obtain signature genes of the old state. The expression of these signature genes over the five age groups is shown in Figure 4a–d(i), respectively. The genes in Figure 4a,d are highly expressed in young individuals and get downregulated during aging, whereas the ones in Figure 4b,c are lowly expressed in young individuals and get upregulated during aging.

For each of these four gene sets, we created an intermingling difference map (see Figure 4a–d(ii), respectively). Figure S15 provides a view of these difference maps zoomed in on age‐specific intermingling contacts, colored according to the magnitude of LAS score differences between the young and old cell states (see Methods). Next, we further quantified the amount of young‐specific and old‐specific intermingling. The signature DE genes that get downregulated during aging have more young‐specific than old‐specific intermingling (Figure 4a,d(iii), and Figure S16), whereas the DE genes that get upregulated during aging have significantly more old‐specific than young‐specific intermingling (Figure 4b,c(iii), and Figure S16). We note that DE genes that get downregulated during aging experience more intermingling changes between the young and old condition than DE genes that get upregulated during aging. Taken together, this means that the selected signature DE genes have more intermingling interactions with each other in the cell state in which they are higher expressed. This observation suggests the formation of transcription hubs in which these sets of genes get co‐activated or co‐repressed in a spatiotemporal manner.

### 2.5 Hi‐C analysis highlights intermingling differences between the gene targets of key bridge TFs

**FIGURE 5:**
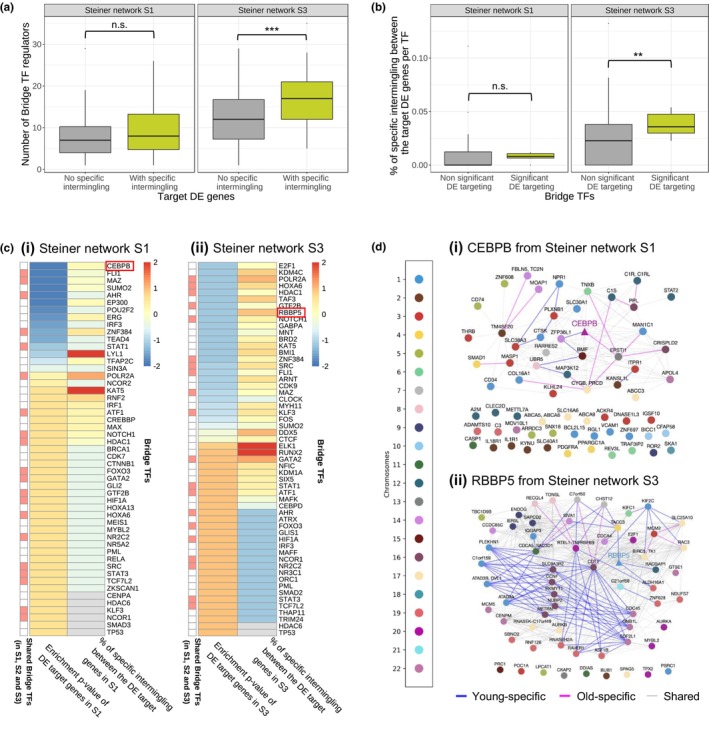
Hi‐C analysis highlights intermingling differences between the gene targets of key bridge TFs. (a) Distribution of the number of bridge TFs included in the corresponding Steiner network targeting the target DE genes in Steiner network S1 (left) and S3 (right). The target DE genes per network were grouped into those whose intermingling status with other target DE genes changes with aging (green box) and the ones whose intermingling does not change with aging (grey box). (b) Distribution of the amount of specific intermingling for significant bridge TFs (green box) and non‐significant bridge TFs (grey box) from Steiner network S1 (left) and S3 (right). Significant bridge TFs correspond to the TFs in Figure 2f and the intermingling change for a bridge TF is defined as the percentage of intermingling interactions changing between its target DE genes. An intermingling interaction change refers to a pair of genes that is only part of an LAS submatrix in young or old Hi‐C data, but not in both. (c) Heatmap with hierarchical clustering of the bridge TFs (y‐axis) in the Steiner network S1 (c(i)) and S3 (c(ii)) for two features (x‐axis) using the Euclidean metric and complete linkage. The selected features are (i) the *p*‐value for enrichment in DE gene targeting from the hypergeometric test (Figure S10; Figure 2f(ii)) the proportion of intermingling interactions between the target DE genes in the corresponding network targeted by a TF that were only part of an LAS submatrix in young or old Hi‐C data, but not in both. All values were *z*‐scored for each feature and clipped to [−2, 2]. The annotation bar on the left side of the heatmaps marks bridge TFs included in all three Steiner networks (S1, S2 and S3) in red. Grey entries correspond to NAs as some bridge TFs have no targets among the upregulated target DE genes in S1 or the downregulated target DE genes in S3. (d) Intermingling network of CEBPB and its upregulated DE target genes in S1 (d(i)) and RBBP5 and its downregulated DE target genes in S3 (d(ii)). Gene–gene interactions that were only part of an LAS submatrix in young Hi‐C data are shown as blue edges, those only found in old Hi‐C data are shown as magenta edges, and the shared ones as grey edges. Nodes are colored according to the chromosome on which the gene is located. The bridge TF nodes are shaped as triangles. The unconnected nodes correspond to DE genes targeted by CEBPB, or RBBP5 respectively, that do not have any intermingling with the other DE genes targeted by this TF.

To connect the key aging‐related TFs obtained in the prize‐collecting Steiner tree analysis to the identified changes in spatial gene clustering during aging, we analyzed the intermingling of the target DE genes in the earliest and latest Steiner networks (S1 and S3, given that the Hi‐C data were from a 10‐year‐old and a 75‐year‐old individual). As most target DE genes in the Steiner network S1 were upregulated (Figure S3), we focused on the upregulated genes for this analysis. On the contrary, as most target DE genes in S3 were downregulated, we focused on the downregulated genes for this analysis. For these two groups of genes, an intermingling difference matrix was created, and the genes were grouped as in Figure 4a(ii) into the ones that change their intermingling between the young and old condition (green) and the ones that do not have intermingling changes (grey). We found that the upregulated target DE genes in S1 and the downregulated target DE genes in S3 that had age‐associated intermingling changes were targeted by more bridge TFs compared to the target DE genes without age‐associated intermingling changes (Figure 5a); this finding was particularly significant in S3 (right panel of Figure 5a, *p*‐value = 1.4e‐6, one‐sided Welch *t* test).

Additionally, we quantified, for each bridge TF, by how much the intermingling between its target genes changes with aging. For this, we created an intermingling difference map for each bridge TF including the upregulated target DE genes in S1 or downregulated target DE genes in S3. In these maps, the percentage of entries with cell‐state‐specific intermingling (entries that were only part of an LAS matrix in the young or the old Hi‐C data) was calculated and compared between the group of bridge TFs that are enriched in targeting DE genes (Figure 2f) and all other bridge TFs. This analysis showed that the target DE genes of significant bridge TFs tend to have more age‐associated intermingling changes (Figure 5b); this result was again found to be particularly significant in S3 (right panel of Figure 5b, *p*‐value = 0.002, one‐sided Welch *t* test).

These results suggest an interplay between gene regulatory programs targeted by key bridge TFs of the aging process and the spatial organization of the genes involved in these regulatory programs. Interestingly, this interplay is substantially weaker or absent at the level of non‐DE target genes of the bridge TFs and at the level of age‐associated DE genes that are not targeted by any bridge TF (Figure S18).

Next, we visualized the changes in the network of Hi‐C contacts between a TF and its target genes for the significant bridge TFs with the highest percentage of age‐associated changes in the intermingling of their target genes (CEBPB in the Steiner network S1 and RBBP5 in the Steiner network S3). This metric, along with the target enrichment *p*‐value corresponding to each TF, are reported in the *z*‐scored heatmap for all bridge TFs in S1 (Figure 5c(i)) and S3 (Figure 5c(ii)); for additional features for each TF such as the number of target genes or the number of protein–protein interactions (PPIs) in the Steiner networks see Figure S19. The selected TFs are interesting because they are key to bridging the DE genes between the young and old conditions and their gene targets undergo 3D organizational changes during aging. The corresponding networks are shown in Figure 5d and Figures S20 and S21. The subnetwork associated with CEBPB consists of 66 nodes and 214 intermingling interactions (edges), out of which 8.4% are young‐specific and 12.1% are old‐specific. Interestingly, CEBPB has two intermingling interactions with its target genes that were only found in young or old cells (young‐specific = blue edges, old‐specific = magenta edges in Figure 5d(i)). CEBPB has been described in a previous study as a key regulator of energy metabolism and longevity (Xia et al., 2021). In the subnetwork associated with RBBP5, 14.2% of the 1084 edges between the 62 nodes are young‐specific and 1.5% are old‐specific, with RBBP5 having three young‐specific intermingling interactions with its target genes (Figure 5d(ii)). This high amount of young‐specific intermingling between the downregulated targets of RBBP5, which has been associated to the age‐driven loss of H3K4 methylation (Yuan et al., 2015), supports the hypothesis that these genes might be co‐activated in the young state and are less expressed in the old state, potentially due to the loss of spatial clustering between them.

## 3 DISCUSSION

The work presented in this study offers a systematic analysis of the evolving transcriptional regulatory programs in human skin fibroblasts throughout the aging process. Our analysis shows that aging follows a nonlinear trajectory, characterized by gradual transcriptional changes observed in age groups 1–4 (1–85 years old), followed by an abrupt transcriptional shift toward the end of life in age group 5 (86–96 years old). These results align with previous research suggesting that age‐related genomic and epigenomic alterations occur in spikes rather than a continuous progression (Márquez et al., 2020). We find that the differentially expressed genes between consecutive age groups are involved in biological pathways related to fibroblast function, immune response, and cell cycle (Figure S2D). This finding is consistent with previous studies showing, for example, that the ECM becomes stiffer with age, leading to a more pro‐inflammatory cellular environment (López‐Otín et al., 2023; Uhler & Shivashankar, 2020). Interestingly, our results corroborate recent findings, which identified an age‐associated transcript length imbalance in skin fibroblasts, with longer transcripts exhibiting higher expression levels in older cells (Stoeger et al., 2022).

Several previous studies have acknowledged the significance of TFs and their regulatory interactions in driving the aging process. These studies typically identified TFs based on the expression levels of their target genes, sometimes in conjunction with age‐dependent changes in the expression of genes that code for these TFs (Maity et al., 2022; O'Brown et al., 2015). In order to consider aging as a gradual process and encompass multiple timepoints, our work leverages an innovative Steiner tree methodology that integrates transcriptomic data from different age groups with PPI data and TF‐target data. This integration enables us to identify TFs that could drive the expression changes observed in the subsequent time step. Moreover, our approach also allows the identification of TFs whose expression remains stable throughout the aging process but may still be key to the aging process because of changes in cellular architecture and 3D chromatin organization, leading to alterations in their cellular compartmentalization or the accessibility of their target genes. The high level of self‐ and cross‐regulation among the identified TFs suggests that aging is highly programmed and self‐reinforcing. For example, we identified E2F1, a known regulator of cellular senescence, as a bridge TF using our Steiner tree analysis.

Beyond the transcriptional lens, we delved into the relationship between age‐associated changes at the transcriptional level and changes in chromatin organization. While most of the literature on 3D chromatin organization has focused on intrachromosomal contacts (Maass et al., 2019), we focused on interchromosomal contacts, as we observed these to exhibit significant changes between the young and the old conditions. Using chromosomal conformation capture data from two time points (10 years old and 75 years old), we found that genes upregulated in young cells exhibited increased intermingling among each other within the nucleus of young cells compared to old cells, and vice versa. Furthermore, we observed that the target genes of the key age‐associated TFs identified using our Steiner tree approach displayed enhanced intermingling compared to other TF target genes. Thus, our Hi‐C data provide strong experimental validation of the identified bridge TFs through our network‐based approach. These observations are consistent with the existence of interchromosomal functional gene clusters implicated in key cell state‐specific processes (Belyaeva et al., 2017; Chen et al., 2015; Papantonis & Cook, 2013; Uhler & Shivashankar, 2017). Interestingly, our analysis uncovered specific TFs whose target genes are differentially expressed through aging and experience age‐specific interactions through chromosomal intermingling. These TFs include CEBPB, known for its role in energy metabolism and longevity (Xia et al., 2021); AHR, recently associated to vascular and brain age‐related phenotypes (Eckers et al., 2016; Ojo & Tischkau, 2021); ERG, linked to fibrosis in mice via its role in chromatin remodeling (Caporarello et al., 2022); STAT1, associated with transcriptional changes and inflammation in the aging human kidney (O'Brown et al., 2015); RBBP5, potentially associated to the age‐driven loss of H3K4 methylation (T. Yuan et al., 2015); BMI1, which was shown to protect hematopoietic stem cells against aging (Nitta et al., 2020); HDAC1, which plays a critical role in aging of the liver and fibroblast senescence through histone deacetylation (Willis‐Martinez et al., 2010), among others.

Taken together, our results provide a systematic characterization of age‐associated transcriptional regulatory programs in human skin fibroblasts, with a particular focus on elucidating the key TFs that could drive the progression of these programs during aging. Importantly, we identified a tight coupling between changes in transcription and spatial gene clustering during aging. These findings highlight the importance of systematically evaluating paired time course transcriptional and chromatin conformation data to advance our fundamental understanding of aging. Similar to recent work identifying rejuvenating TFs (Sengstack et al., 2022), we anticipate that some of the TFs we identified in this work could serve as potential therapeutic targets to attenuate, prevent, or even reverse age‐related declines, offering life‐changing benefits to the growing global geriatric population.

## 4 METHODS

### 4.1 RNA‐Seq data

Transcriptomic data were retrieved from a previous study (Fleischer et al., 2018), which generated bulk RNA‐Seq measurements of skin fibroblasts for 133 individuals between 1 and 96 years old. The corresponding FASTQ files containing the sequencing information were downloaded from the European Nucleotide Archive with accession numbers SRR7093809 to SRR7093951. For transcript quantification, the tool Salmon (Patro et al., 2017) was used. The sequenced reads were aligned to the *Homo sapiens* reference transcriptome from Ensembl release 105. Then, Salmon was used to create an index on that reference transcriptome, as well as a quant.sf file for each sample, which contains the count values per transcript. The transcript counts per sample were then imported into R with tximeta (Love et al., 2020). Additionally, two different normalization techniques were applied to the raw counts. First, for gene expression comparisons within a sample, the Fragments Per Kilobase of transcript per Million mapped reads (FPKM) values for each gene and for each individual were calculated using the R package DESeq2. Second, for gene expression comparisons between samples, scaling factors for library‐size correction were applied and the variance‐stabilized transformed counts were obtained using DESeq2. Given the strong correlation between mean and variance of the expression of a gene, we used variance‐stabilizing transformation on the counts to make the variance independent of the mean. This is useful given that many downstream statistical methods assume homoscedastic data (Anders & Huber, 2010). To define the activity of each gene, the histogram of the logarithmic mean FPKM value per age group was plotted, showing a bimodal distribution (Figure S5A). A threshold of 0.8 on the FPKM values was chosen as this marks the end of the first mode. In each age group, genes with a mean expression below that threshold were considered inactive and the remaining genes were considered active. Over 11,000 genes were found to be active in all five age groups, but there were also more than 800 genes that were only active in a subset of the age groups (Figure S5B).

### 4.2 PPI data

A network with proteins as nodes and edges corresponding to direct, physical PPIs in *Homo sapiens* was obtained from the STRING database version 11.5 (Szklarczyk et al., 2019). We decided to use STRING (Bajpai et al., 2020) in this study because it is among the databases that have the highest coverage of experimentally verified protein interactions and it also allows to select only physical interactions. The reported annotations in STRING refer to the Ensembl peptide IDs, while the networks were built using gene names. Therefore, the annotation file provided on the STRING website was used for renaming. As some of the peptide IDs correspond to the same gene name, the dataset included 17,954 distinct proteins after renaming of the 18,384 peptide IDs and 1,900,568 physical interactions between pairs of proteins. In the STRING database each interaction has a confidence score of being present based on its evidence in the literature. The scores for physical interactions range from 0 to 1000, in which high numbers mean that there were many experiments which report a physical interaction between two proteins (Szklarczyk et al., 2019). We transformed these edge confidence scores into edge costs so that they can be used in the prize‐collecting Steiner tree algorithm, which is formulated as a minimization problem over edge costs. We chose to reverse the confidence scores x into costs between 0 and 1 using the following formula: cost(*x*) = 1 – (*x* – min_score)/(max_score – min_score) with min_score referring to the lowest score and max_score to the highest score in the dataset. A pseudo‐count of 0.01 was added to prevent having a cost of zero; the distribution of the costs over all interactions is shown in Figure S4A.

### 4.3 Transcription regulator – Target data

The database hTFtarget was used to obtain information about which human TFs target which genes (Zhang, Liu, et al., 2020). This database contains data from 7190 large‐scale Chromatin Immuno‐Precipitation sequencing (ChIP‐Seq) experiment samples of 659 TFs in 569 different conditions, including a broad range of cell types. Only 48 out of 659 TFs reported in hTFtarget had targets that were specifically reported for fibroblast cells in various tissue types. Therefore, the TF‐target interactions over all cell types were used even though this might include some false positive interactions that are not present in fibroblast cells due to cell‐type‐specific TF‐target binding. We took this approach, given that gene expression values are considered during network construction and thus missing TF‐target links are more harmful than the addition of false edges. Therefore, we downloaded a file from hTFtarget that reports all TF‐target links which were found in more than 30% of ChIP‐Seq datasets. This file includes 1319,123 regulatory links between 495 TFs and 38,183 targets. The list of transcription regulators and target genes was then filtered to the ones included in STRING. Most of the TFs only have a few target genes (Figure S4B).

### 4.4 Chromosomal conformation data

Low input in‐nucleus Hi‐C data were obtained from the GEO database under accession number GSE237271 (Sornapudi et al., 2023). The dataset consists of two biological replicates of (young) healthy human dermal fibroblasts derived from a 10‐year‐old donor (GM09503), as well as two biological replicates of (old) healthy human dermal fibroblasts derived from a 75‐year‐old donor (GM08401). Each biological replicate includes approximately 200,000 cells. As we had access to the mapped sequencing reads in the .pair format, the first step was to convert the files to the .hic file format using Juicer tools software with a resolution of 250 kb and a mapping quality threshold of 30 (Durand et al., 2016). Chromosomes X and Y were not considered in this study. As Hi‐C data may contain locus‐specific multiplicative biases which depend, for example, on the accessibility of a locus, a matrix‐balancing normalization was performed that balances the Hi‐C map into a doubly stochastic matrix. For the intrachromosomal contact maps the “SCALE” normalization from Juicer tools was used (Durand et al., 2016). For interchromosomal contacts, we used the “INTERSCALE” normalization from Juicer tools which produces a doubly stochastic matrix for the genome‐wide Hi‐C map after removing all intrachromosomal maps. Additionally, there exist chromosome regions like repeats, centromeres or pericentromeric regions (2 Mb in both directions starting at the centromere), where mapping of sequencing reads is challenging due to their repetitiveness. Therefore, these regions were excluded from the analysis using the annotation from the UCSC table browser for reference genome GRCh38 (Karolchik et al., 2004). Finally, a log(*x* + 1) transformation was applied to the normalized Hi‐C contact maps. We also created a *z*‐scored version of the data. In this version, the mean and standard deviation over all intrachromosomal contacts was calculated, as well as the mean and standard deviation over all interchromosomal contacts. Then, *z*‐scores were obtained by subtracting the corresponding mean and dividing by the corresponding standard deviation. The distribution of the *z*‐scored inter‐ and intrachromosomal values in the two young and two old replicates are shown in Figure S14B. We used the *z*‐scored version of the Hi‐C data when running the LAS algorithm. Note that we used Hi‐C data for GM09503 (young) and GM08401 (old) fibroblast cells to study the 3D organization of age‐associated DE genes obtained from bulk RNA‐Seq of skin fibroblasts from research subjects (Fleischer et al., 2018). To test that these cell types are similar and can be used in a combined analysis, we correlated the transcriptional profiles of GM09503 cells with cells from individuals in age group 1 (correlation coefficient *R* = 0.96, *p*‐value <2.2e‐16) and the transcriptional profiles of GM08401 with age group 5 (*R* = 0.95, *p*‐value <2.2e‐16), resulting in highly significant correlation coefficients; see Figure S17.

### 4.5 Definition of age groups

A hierarchical clustering approach was used to define five age groups. For this clustering approach, we used a subset of genes that were found to be associated with age in a linear regression model. First, LASSO regression was performed with age as the predicted variable and gene counts as predictors. We tested five LASSO penalty coefficients (*λ* ∈ {0.25, 0.5, 1, 2, 4}). The higher the penalty coefficient, the fewer genes have a nonzero coefficient. A medium penalty coefficient of *λ* = 1 was chosen, which results in 101 genes with a nonzero coefficient. Next, for hierarchical clustering, a distance matrix of all individuals was created by calculating the Euclidean distance between two individuals based on the variance‐stabilized expression of genes selected by the LASSO regression. Then, the distance matrix was used for hierarchical clustering with the Ward agglomeration method (ward.D2) using the pheatmap package. The dendrogram was cut to obtain 6 clusters (phenetic threshold = 17) and the age distribution per cluster was visualized as a boxplot (Figure 1d). We chose six clusters because increasing the number of clusters did not improve the age separation. As the age range of two of the groups were overlapping, only five age groups were defined based on the obtained clusters: Group 1 (1–15 years old), Group 2 (16–26 years old), Group 3 (27–60 years old), Group 4 (61–85 years old), and Group 5 (86–96 years old).

### 4.6 Differential gene expression analysis

To identify key genes related to the transitions between consecutive age groups, a differential gene expression analysis was performed. For this, the R package DESeq2 was used on the raw RNA‐Seq counts obtained from tximeta. Typically, a threshold for the FDR‐adjusted *p*‐value from a Wald test is used to distinguish between DE and non‐DE genes. In addition to this threshold, we used a robustness analysis to assure that a gene is consistently found as DE when subsampling the individuals from the dataset (Fleischer et al., 2018). This procedure dampens the effect of outlier values in single individuals. 100 simulations were performed in which 80% of the individuals per age group were randomly selected and the DE genes (FDR‐adjusted *p*‐value <0.1) per transition from one age group to the next were recorded. Only genes (i) having a significant DE *p*‐value, (ii) identified in at least a certain proportion of the subsamples, and (iii) having a high log2‐fold change value were selected as the final DE genes as follows. First, transition‐specific *p*‐value thresholds were applied to select approximately 400 genes per transition. Second, transition‐specific robustness thresholds were chosen based on the transition's robustness profile (see Figure S2A) to discard non‐robust genes. Finally, transition‐specific log2‐fold change thresholds were used to select approximately 160–180 DE genes per transition. These three combined levels of thresholding (shown in Figure S2C) guarantee the selection of a robust and meaningful set of DE genes for each age group transition; see also the GO analysis in Figure S2D.

### 4.7 Protein network design

Three networks (N1, N2, and N3) corresponding to different life stages were built. For N1, we filtered the STRING interactome to proteins corresponding to actively expressed genes in Group 1 or Group 2 and prized the DE genes between Group 1 and Group 2 (source DE genes) by their absolute log2 fold change. Additionally, nodes for the DE genes in the consecutive transition (Group 2 vs. Group 3) were added (target DE genes) and prized by their log2 fold change. To connect the PPI network part to the target DE genes, edges from bridge TFs in the PPI network were added to target DE genes that are targeted by these TFs based on TF‐target interactions from the data base hTFtarget. A cost of 0.01, which corresponds to the minimum cost for the PPIs, was assigned to these TF‐target edges. In N2, Group 2 and Group 3 were used for the source DE genes and Group 3 and Group 4 for the target DE genes, and in N3, Group 3 and Group 4 were used to define the source DE genes and Group 4 and Group 5 for the target DE genes. Five different network design choices were tested and compared for robustness, in which the first is the one described above. In Design 2, information from the oldest subnetwork (S3) was used for constructing the next younger one (S2), as well as from S2 to S1 in the following way: in addition to prizing DE genes, bridge TFs included in the next‐older subnetwork were added to the target DE genes of the younger one. They were connected by edges to bridge TFs in the PPI and their nodes were prized with the minimum prize over the target DE genes. The intuition behind this approach is to ensure that also bridge TFs that target bridge TFs driving the aging process in the next life stage are identified. For Design 3 and onward, we also added PPI edges between the target DE gene nodes (red nodes in Figure 2a). In Design 4, not only bridge TFs, but also all other nodes in the PPI network of the next older subnetwork, as well as their PPIs with the target DE genes were added. Finally, in Design 5, the unprized nodes from the next older network were not only included, but also prized with the minimum prize of the target DE genes. To compare the results of the different design choices, the prize‐collecting Steiner tree algorithm was used to identify a smaller subnetwork including the prized genes. As there was a high overlap between the bridge TFs that were included across the design choices (Figure S7), Design 2 was selected and used for all subsequent analyses.

### 4.8 Null distributions for significance testing

In Figure 2d, TF‐target links from hTFtarget were used to build regulatory networks between groups of TFs. To assess whether there are significantly more edges in the networks of Figure 2d than expected by chance, a null distribution was obtained by doing 1000 simulations selecting 20 random bridge TFs that were included in at least one of the three Steiner networks. For each of the simulations, the regulatory network was created. For each of the four networks containing the bridge TFs from one of the four groups, as well as for the 1000 networks with randomly selected bridge TFs, the network density defined as (number of edges)/(number of nodes) was measured. The numerical *p*‐value for the network in each of the four groups was calculated as the percentage of simulated networks whose density was at least as high as the observed network density of the group under consideration.

In Figure 2e, we show which TF pairs have significantly more intersecting targets than expected under a hypergeometric null model. This figure was constructed in the following way. Out of all N genes in the genome, N_1_ genes are targeted by the first of the two TFs. The hypergeometric distribution then describes for the second TF with N_2_ target genes, how likely it is to observe k intersecting targets. A *p*‐value for each TF pair was obtained by using the cumulative distribution function calculating the probability of observing at least *k* intersecting targets with the function stats.hypergeom.sf (*k*‐1, N_1_, N_2_, N) from the Python package scipy. Additionally, the proportion of shared targets was calculated as |{targets of TF_1_} ∩ {targets of TF_2_}|/|{targets of TF_1_} ∪ {targets of TF_2_}| for each pair of bridge TFs (TF1, TF2).

To evaluate which of the bridge TFs in the Steiner networks target significantly more target DE genes than genes in the genome in general, a hypergeometric null model was used (see Figure 2f and Figure S9). The hypergeometric distribution has four parameters, namely the total number of genes (*M*), the total number of DE genes (*n*), the number of target genes of a bridge TF in the genome (N), as well as the number of DE genes that are targeted (*k*). The *p*‐values were calculated using the function stats.hypergeom.sf(*k*‐1, *M*, *n*, N) from the Python package scipy. The *p*‐values were adjusted for multiple testing using Bonferroni correction. Additionally, 95% confidence intervals were calculated over multiple values of N (the number of target genes in the genome).

To evaluate whether there is significantly more or less intermingling between the selected genes in Figure 4 compared to random ones, 1000 simulations were performed sampling the same number of random genes. In each intermingling difference map, the percentage of young‐specific, old‐specific, and shared intermingling was calculated. Based on these null distributions, *p*‐values were calculated as the percentage of simulations with a higher percentage for each intermingling type (Figure S16).

### 4.9 Data and code availability

RNA‐Seq data were obtained from the European Nucleotide Archive with accession numbers SRR7093809–SRR7093951. For network construction, we used protein interactions reported in the STRING database version 11.5 (Szklarczyk et al., 2019) and TF‐target interactions from the database hTFtarget (Zhang, Liu, et al., 2020). The Hi‐C data used in this study can be obtained from the GEO database under accession number GSE237271.

The code to reproduce the analysis and figures in this work can be found on GitHub (https://github.com/uhlerlab/aging_transcriptome_and_chromatin). Our analyses used R version 4.2.1 with the tidyverse package (version 1.3.2). To read in the RNA‐Seq data into R, we used the package tximeta (version 1.14.1). The package DESeq2 (version 1.36.0) (Love et al., 2014) was used to call differentially expressed genes. The package biomaRt (version 2.52.0) was used to get the genomic location of each gene (Durinck et al., 2009) and in case a gene spans multiple 250 kb loci of the Hi‐C data, only the first one in genomic order was used. To create intersection plots, we used the package UpSetR (version 1.4.0) (Conway et al., 2017). To create hierarchically clustered heatmaps, we used the package pheatmap (version 1.0.12).

We also conducted analyses using Python version 3.7.13. Network analyses and visualizations were conducted in Python using the package networkx (version 2.4) (Hagberg et al., 2008) and the package OmicsIntegrator 2 (version 2.3.10) (Huang & Fraenkel, 2009). Gene ontology (GO) analyses were performed with the enrichr function of the Python package gseapy (version 0.10.8) (Fang et al., 2023) with the gene set “GO_Biological_Process_2021.” For hypergeometric testing and *p*‐value computations, we used the package scipy (version 1.7.3). The function stats.pearsonr from scipy was used to calculate the Pearson correlation coefficient and the *p*‐value for testing non‐correlation. The package upsetplot (version 0.6.1) was used for the intersection plot in Figure 3b. Hi‐C distance decay profiles, insulation scores, and boundary scores were computed using the package fanc (version 0.9.27) (Kruse et al., 2020).

For visualization, *p*‐values were encoded using the following convention: *p*‐value <0.05 (*), *p*‐value <0.01 (**), *p*‐value <0.001 (***).

## AUTHOR CONTRIBUTIONS

Conceptualization: JMB, LVC, CU, and GVS; methodology: JMB, LVC, TRS, CU, and GVS; software: JMB and LVC; formal analysis: JMB, LVC, TRS, CU, and GVS; writing: JMB, LVC, CU, and GVS.

## FUNDING INFORMATION

This work was partially supported by the Eric and Wendy Schmidt Center at the Broad Institute, NCCIH/NIH (1DP2AT012345), ONR (N00014‐22‐1‐2116), AstraZeneca, and a Simons Investigator Award to CU, and a Swiss National Foundation award (310030_208046) to GVS. JMB was funded by an Otto Bayer Fellowship for Drug Discovery.

## CONFLICT OF INTEREST STATEMENT

The authors declare no conflict of interest.

## Supporting information


Appendix S1.

## Data Availability

RNA‐seq data was obtained from the European Nucleotide Archive with accession numbers SRR7093809 to SRR7093951. For network construction, we used protein interactions reported in the STRING database version 11.5 and TF‐target interactions from the database hTFtarget. The Hi‐C data used in this study can be obtained from the GEO database under accession number GSE237271.
