# Supplementary material for: Transcriptional changes are tightly coupled to chromatin reorganization during cellular aging
Source: Aging Cell. 2023 Dec 7;23(3):e14056. doi: 10.1111/acel.14056 (PMC10928569; doi:10.1111/acel.14056)
Supplement: Supplementary file 1 — Appendix S1. [file ACEL-23-e14056-s001.pdf]

Supporting Information for

Transcriptional changes are tightly coupled to chromatin reorganization during cellular aging

Jana M. Braunger[1]*, Louis V. Cammarata[1,2]*, Trinadha Rao Sornapudi[3], Caroline Uhler[1,4]† and G.V. Shivashankar[3,5]†

1. Eric and Wendy Schmidt Center, Broad Institute of MIT and Harvard, Cambridge, MA 02139, USA
2. Department of Statistics, Harvard University, Cambridge, MA 02138, USA
3. Division of Biology and Chemistry, Paul Scherrer Institute, 5232, Villigen, Switzerland
4. Laboratory for Information and Decision Systems, Massachusetts Institute of Technology, Cambridge, MA 02139, USA
5. Department of Health Sciences and Technology, ETH Zurich, 8092, Zurich, Switzerland

* Equal contribution

† Corresponding authors: Caroline Uhler (cuhler@mit.edu), G.V. Shivashankar (gshivasha@ethz.ch)

This PDF file includes:

SI Methods
SI Figures S1 to S21

Supplementary Methods

Prize-collecting Steiner tree algorithm

To identify subnetworks in protein networks with physical and regulatory links, we used prize-collecting Steiner forests, which are an extension of Steiner's network problem (Hakimi, 1971). Let $G = (V, E)$ be a connected, undirected graph with non-negative edge costs $c(e)$ for each $e \in E$. Considering a subset of nodes called terminals $S \subset V$, the goal of the classical Steiner problem in graphs is to find a subgraph connecting all terminals with minimum weight. The nodes that are part of that subgraph in addition to the terminals are called Steiner nodes. The prize-collecting Steiner tree (or forest) problem is a generalization where a prize $p(s)$ is given to each terminal $s \in S$ and a dummy node r is added, which corresponds to the root node that is connected to all terminals with a weight of w. The goal is to identify a subnetwork that minimizes the weighted sum of the prizes of the not-included terminals and the costs of the included edge. For implementation, the Forest tool from the Python package OmicsIntegrator 2 (Huang & Fraenkel, 2009) was used, which searches for a connected subgraph $T = (V_T, E_T)$ of the modified graph $G^* = (V \cup \{r\},\ E \cup \{\{r,s\} : s \in S\})$. The corresponding objective function is:

$$\psi(T) = b \cdot \sum_{v \notin V_T} p(V) + \sum_{e \in E_T} c^*(e).$$

The parameter $b \in \mathbb{R}^*$ controls the relative weighting of the node prizes compared to the edge costs. We used $b = 2$ for the experiments to ensure that in most cases all prized proteins are included in the solution. $c^*(.)$ is a modified cost function that takes the degree d_x of a node x in G into account. For any edge $e = \{x, y\} \in E \cup \{\{r,s\}: s \in S\}$:

$$c^*(e) = \begin{cases} c(e) + \dfrac{d_x * d_y}{d_x * d_y + (N - d_x - 1)(N - d_y - 1)} * 10^g & if\ e \in E \\ w \quad if\ e \in \{\{r,s\} : s \in S\} \end{cases}$$

Where $w, g \in \mathbb{R}^+$ are tuning parameters. We used $w = 2$, such that it is at least twice as expensive to include an edge from a terminal to the root node instead of connecting the terminal directly to a node in G. This results in the solution to be a tree and not a forest with multiple unconnected components after the root is removed. The parameter g controls how strongly the edge costs are penalized depending on the node degrees. In general, this adaptation leads to increased costs for nodes that have many edges, which avoids network biases towards `hub nodes', which are highly studied proteins interacting with many others that might not be specific to the biological question of interest (Huang & Fraenkel, 2009). We used $g = 0$ such that this cost scaling is as small as possible, which was shown in previous work (Belyaeva et al., 2021) to give similar results as a setting without any cost-scaling, *i.e.*, $g = -\infty$.

Large Average Submatrix (LAS) algorithm

Due to the noise that corrupts Hi-C data matrices, a pixel-by-pixel comparison between the young and old samples is not tractable to identify differences in intermingling regions. Therefore, the LAS algorithm, a bi-clustering procedure which searches for

continuous submatrices $U \in \mathbb{R}^{k \times l}$ with a high average value τ in a real-valued data matrix $X \in \mathbb{R}^{m \times n}$, was used to call intermingling regions (Shabalin et al., 2009). LAS is an iterative, heuristic algorithm that scores submatrices based on a trade-off between matrix size and average value. The LAS algorithm was run for all chromosome pairs of chromosomes 1-22 as described in (Belyaeva et al., 2017) with a maximum allowed matrix size of 10 Mb × 10 Mb, *i.e.*, 40 × 40 pixels in the Hi-C maps with a resolution of 250 kb. The threshold was determined based on a *p*-value of 1e-15 by using the distribution over all Hi-C entries of inter- and intrachromosomal contacts separately (Supplementary Fig. 14B), thereby controlling the FWER at approximately 1e-4. This resulted in a score threshold of 20 for the interchromosomal contact maps and a score threshold of 5 for the intrachromosomal maps, where the LAS submatrix score is defined as $\sqrt{\mathrm{k}}\tau$ for a submatrix of size k-by-k. Since the LAS algorithm is not guaranteed to find all significant submatrices and it could happen that a submatrix is found in one sample, but not in the other even though the submatrix score would be above the threshold there too, for all LAS submatrices that were identified in at least one sample, we calculated the corresponding score in all other samples. The results for the two replicates per condition were combined by only keeping submatrices whose score was higher than the threshold in both replicates.

Bibliography

Belyaeva, A., Cammarata, L., Radhakrishnan, A., Squires, C., Yang, K. D., Shivashankar, G. V., & Uhler, C. (2021). Causal network models of SARS-CoV-2 expression and aging to identify candidates for drug repurposing. *Nature Communications*, *12*(1), 1024.

Belyaeva, A., Venkatachalapathy, S., Nagarajan, M., Shivashankar, G. V., & Uhler, C. (2017). Network analysis identifies chromosome intermingling regions as regulatory hotspots for transcription. *Proceedings of the National Academy of Sciences*, *114*(52), 13714–13719. https://doi.org/10.1073/pnas.1708028115

Hakimi, S. L. (1971). Steiner's problem in graphs and its implications. *Networks*, *1*(2), 113–133. https://doi.org/10.1002/net.3230010203

Huang, S. C., & Fraenkel, E. (2009). Integrating proteomic, transcriptional, and interactome data reveals hidden components of signaling and regulatory networks. *Science Signaling*, *2*(81), ra40–ra40.

Shabalin, A. A., Weigman, V. J., Perou, C. M., & Nobel, A. B. (2009). Finding large average submatrices in high dimensional data. *The Annals of Applied Statistics*, 985–1012.

Supplementary Figures

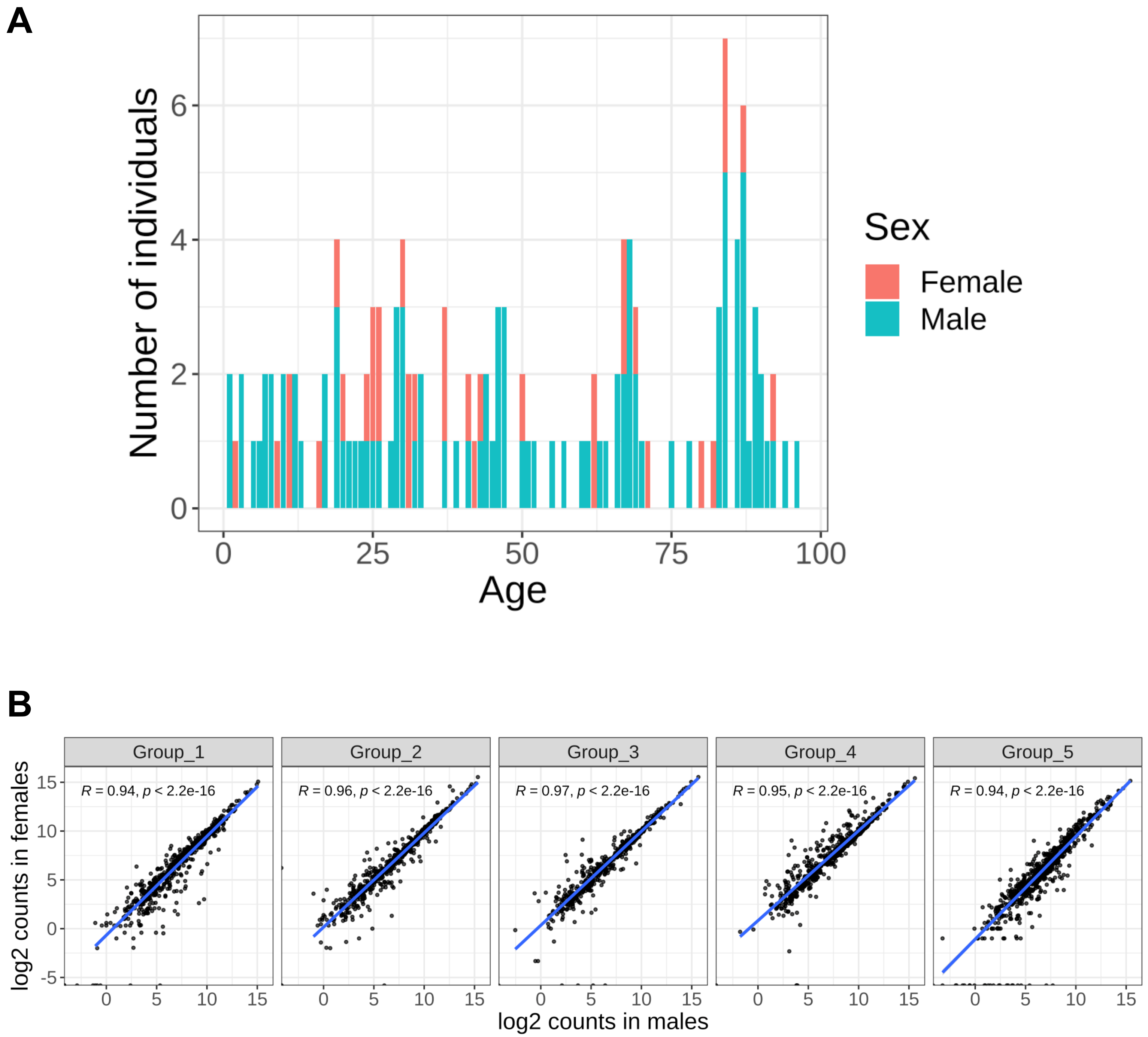

Supplementary Figure 1

Supplementary Figure 1

A. Histogram of the number of individuals for each age in the RNA-seq data set used in this work (see Methods). The distributions are shown for females (red) and males (blue) separately.
B. Gene expression in males (x-axis) vs. females (y-axis) for age-associated DE genes. For each age group, the log2 raw expression counts were averaged across all female and male individuals in the group for each gene that is differentially expressed in at least one age group transition. A fitted linear regression line is shown in blue; the correlation coefficient R and the corresponding *p*-value are reported for each age group.

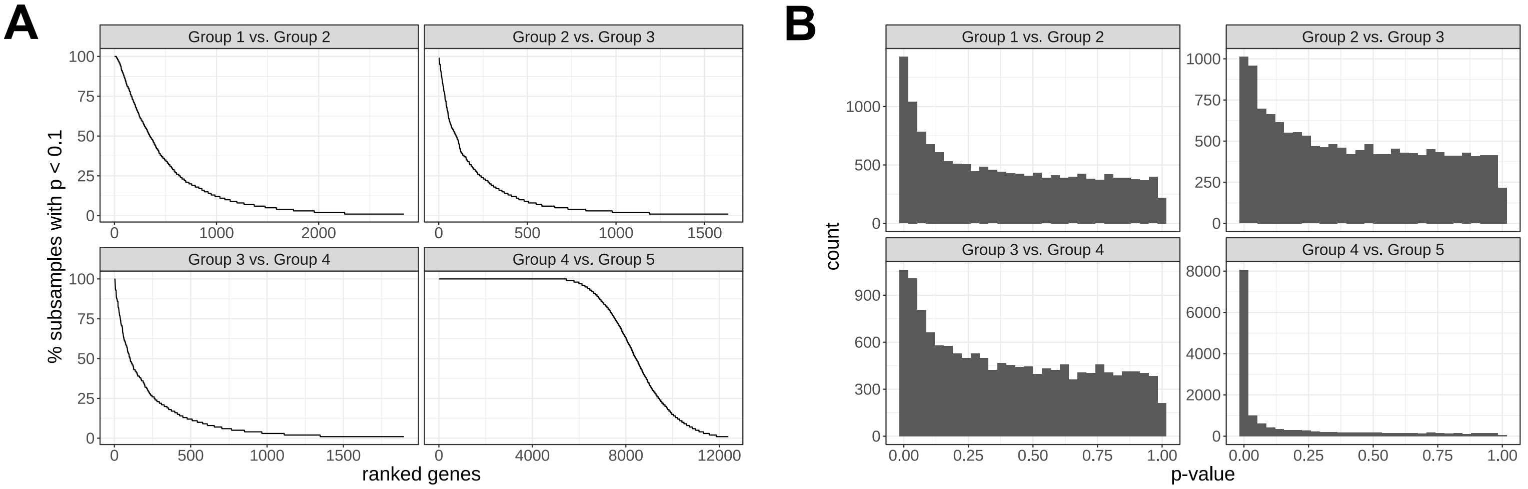

C

Transition	*p*-value threshold	% subsamples threshold	Log2-fold change threshold
Group 1 vs. Group 2	0.05	50	0.6
Group 2 vs. Group 3	0.1	25	0.4
Group 3 vs. Group 4	0.1	20	0.4
Group 4 vs. Group 5	1e-17	99	1.6

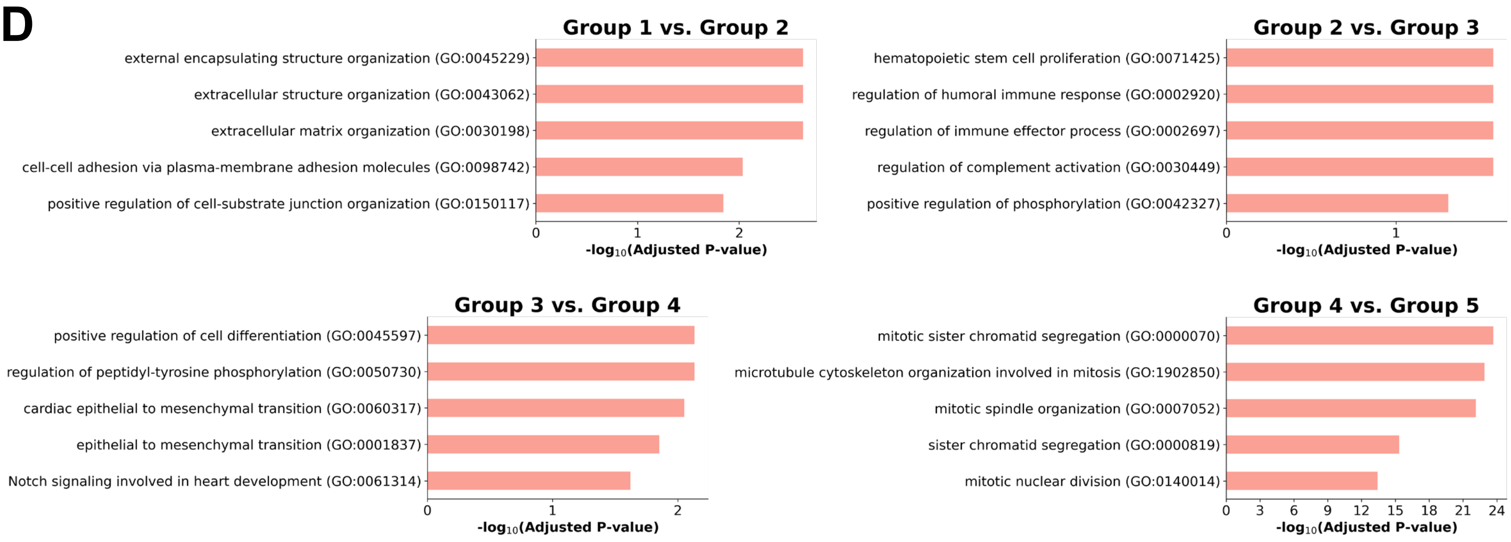

Supplementary Figure 2

Supplementary Figure 2

A. Robustness of DE gene analysis in 100 subsamples containing a random selection of 80% of the individuals per age group. For each subsample, the genes that were differentially expressed between one age group and the next older one (FDR adjusted *p-value* < 0.1) were determined. The percentage of subsamples in which a gene is DE is shown on the y-axis. For each of the four transitions between consecutive age groups, the x-axis contains all genes identified as being DE in at least one of the simulations, sorted in decreasing order of the percentage of subsampling simulations in which a gene occurred as DE.
B. Histogram of the *p*-values obtained for all genes in a differential gene expression analysis between each pair of consecutive age groups.
C. Thresholds to select 160-180 DE genes for each transition between consecutive age groups. 3 types of thresholds were used and adapted for each of the transitions to get approximately the same number of DE genes: (i) the selected DE genes had to have an adjusted p-value lower than the defined threshold, (ii) they had to be included in at least the given percentage of subsamples from A, and (iii) they had to have a log2 fold change higher than the given threshold.
D. Gene Ontology analysis for the selected DE genes for each transition between consecutive age groups. For the four groups of DE genes, the Enrichr gene set 'GO_Biological_Process_2021' was used to obtain functional annotations. The top 5 pathways with the lowest FDR-adjusted *p*-values (*p-value* < 0.05) are shown.

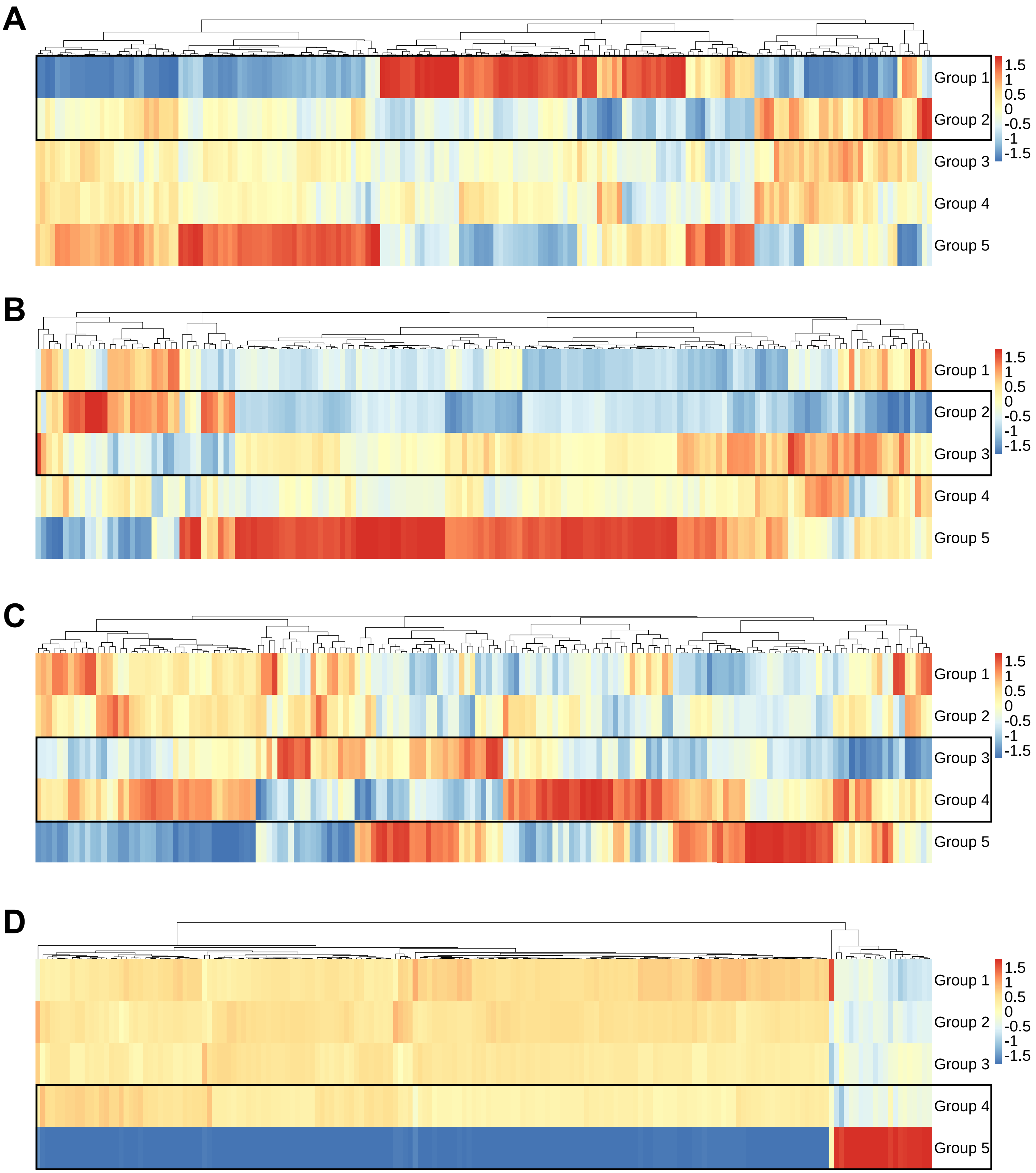

Supplementary Figure 3

Supplementary Figure 3

A. Heatmap of the z-scored RNA-seq expression of the DE genes in the transition from Group 1 to Group 2. The RNA-seq expression is shown for all age groups (Group 1 and Group 2 are boxed in black). The genes (x-axis) are ordered by hierarchical clustering using Euclidean distance with complete linkage.
B. Heatmap of the z-scored RNA-seq expression of the DE genes in the transition from Group 2 to Group 3.
C. Heatmap of the z-scored RNA-seq expression of the DE genes in the transition from Group 3 to Group 4.
D. Heatmap of the z-scored RNA-seq expression of the DE genes in the transition from Group 4 to Group 5.

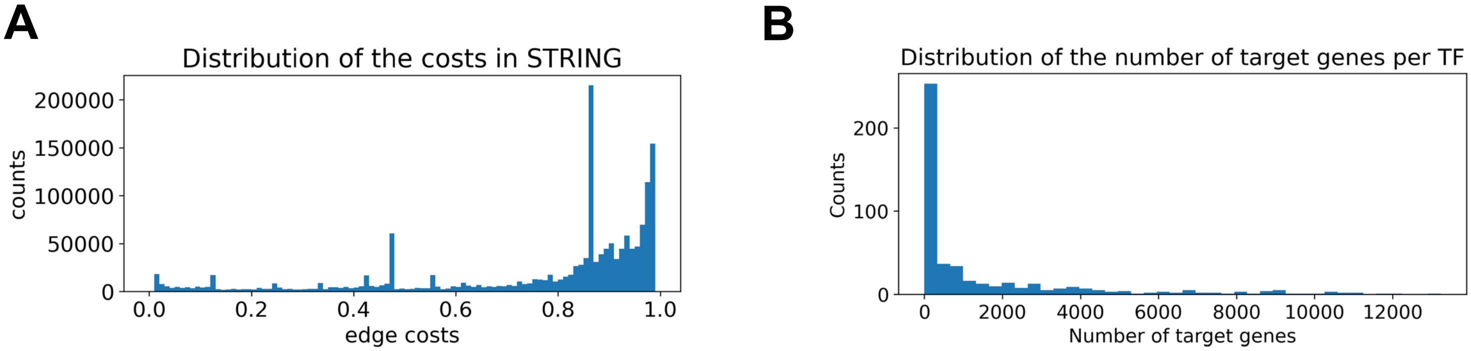

Supplementary Figure 4

Supplementary Figure 4

A. Histogram of the edge cost distribution in the processed protein-protein interaction data retrieved from STRING (see Methods).
B. Histogram of the number of gene targets for the selected TFs retrieved from the hTFtarget database (see Methods).

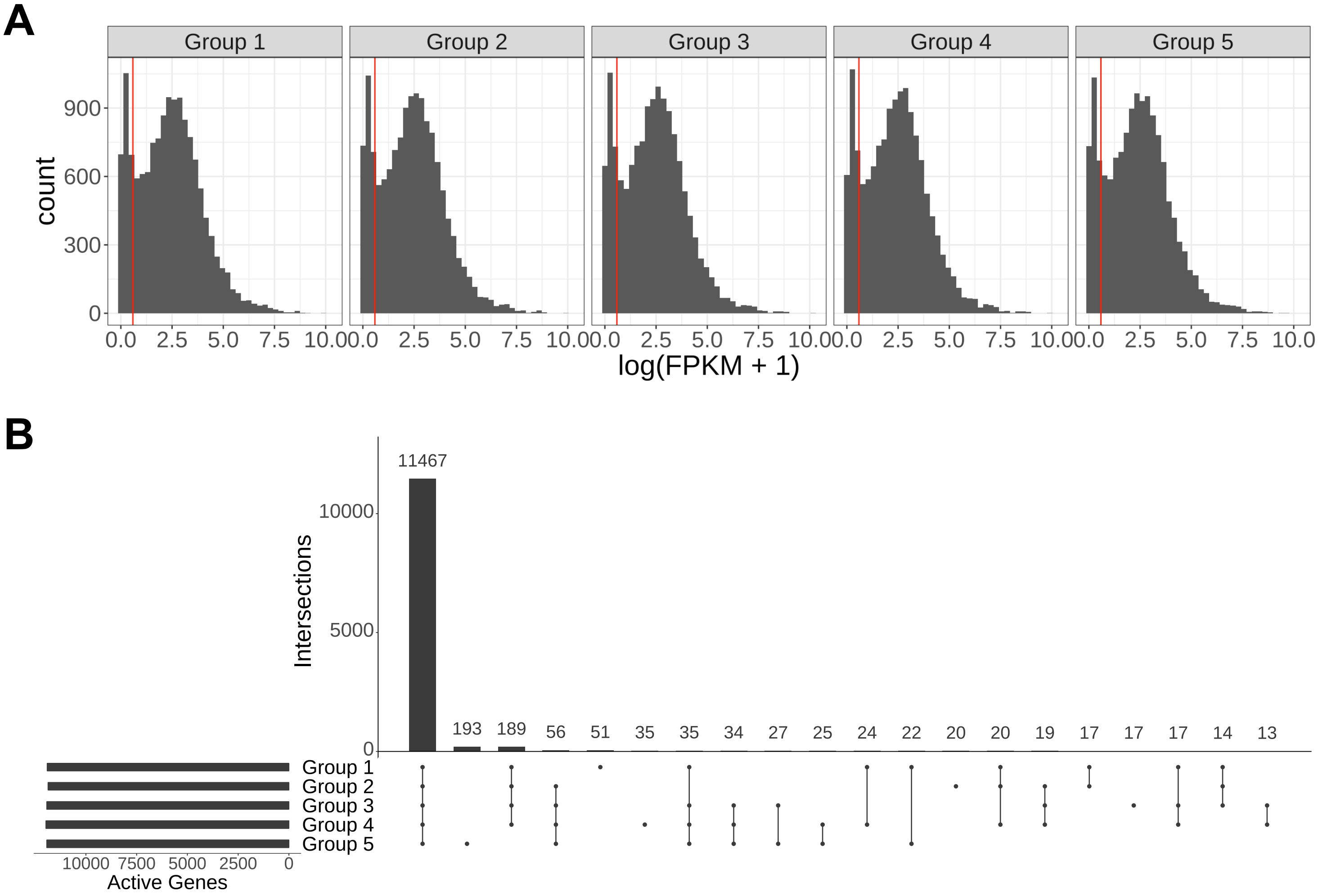

Supplementary Figure 5

Supplementary Figure 5

A. Histograms of the logarithmic mean FPKM gene expression for each age group used to define gene activity thresholds. The threshold (red line, log(FPKM+1) > 0.8) to distinguish between inactive and active genes was set to exclude the first mode. Genes expressed below this threshold were considered inactive, while genes above this threshold were considered active. Only proteins corresponding to active genes were used to construct the Steiner networks.
B. UpSet plot of the active genes in the 5 age groups. While most of the active genes are active in all 5 age groups (11,467 genes), there are also more than 800 genes that change their activity during aging and thus are only active in various subsets of the age groups.

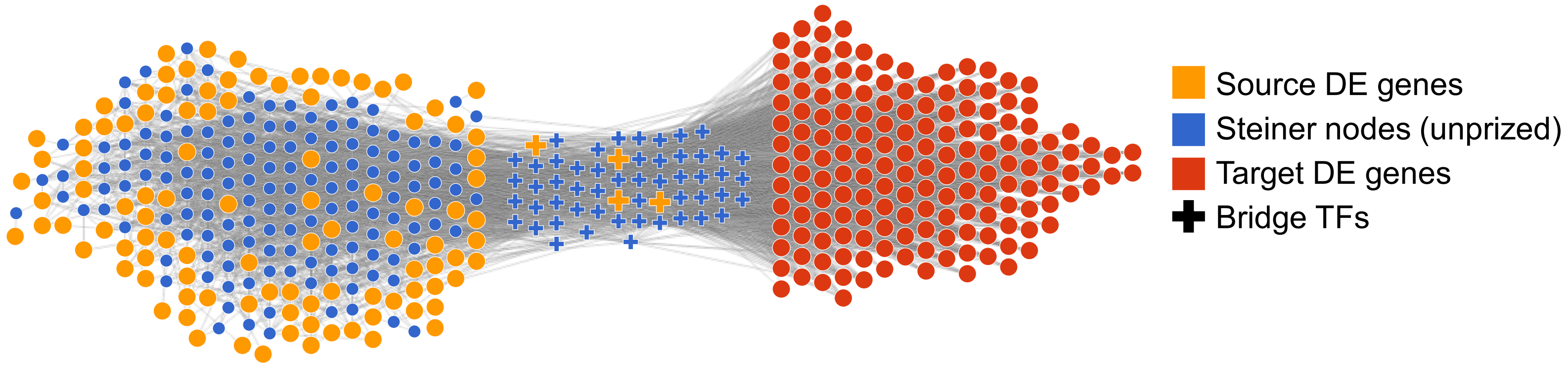

Supplementary Figure 6

Supplementary Figure 6

Prize-collecting Steiner network S3 using the DE genes between Group 3 vs. Group 4 as source DE genes (orange nodes) and the DE genes between Group 4 vs. Group 5 as target DE genes (red nodes). In addition to the DE genes, the network also contains bridge TFs (crosses) and other unprized nodes (Steiner nodes, colored in blue) that help connect the source DE genes to the target DE genes. The nodes were sized according to their prize, which is why the orange and red nodes are in general larger than the blue, unprized nodes.

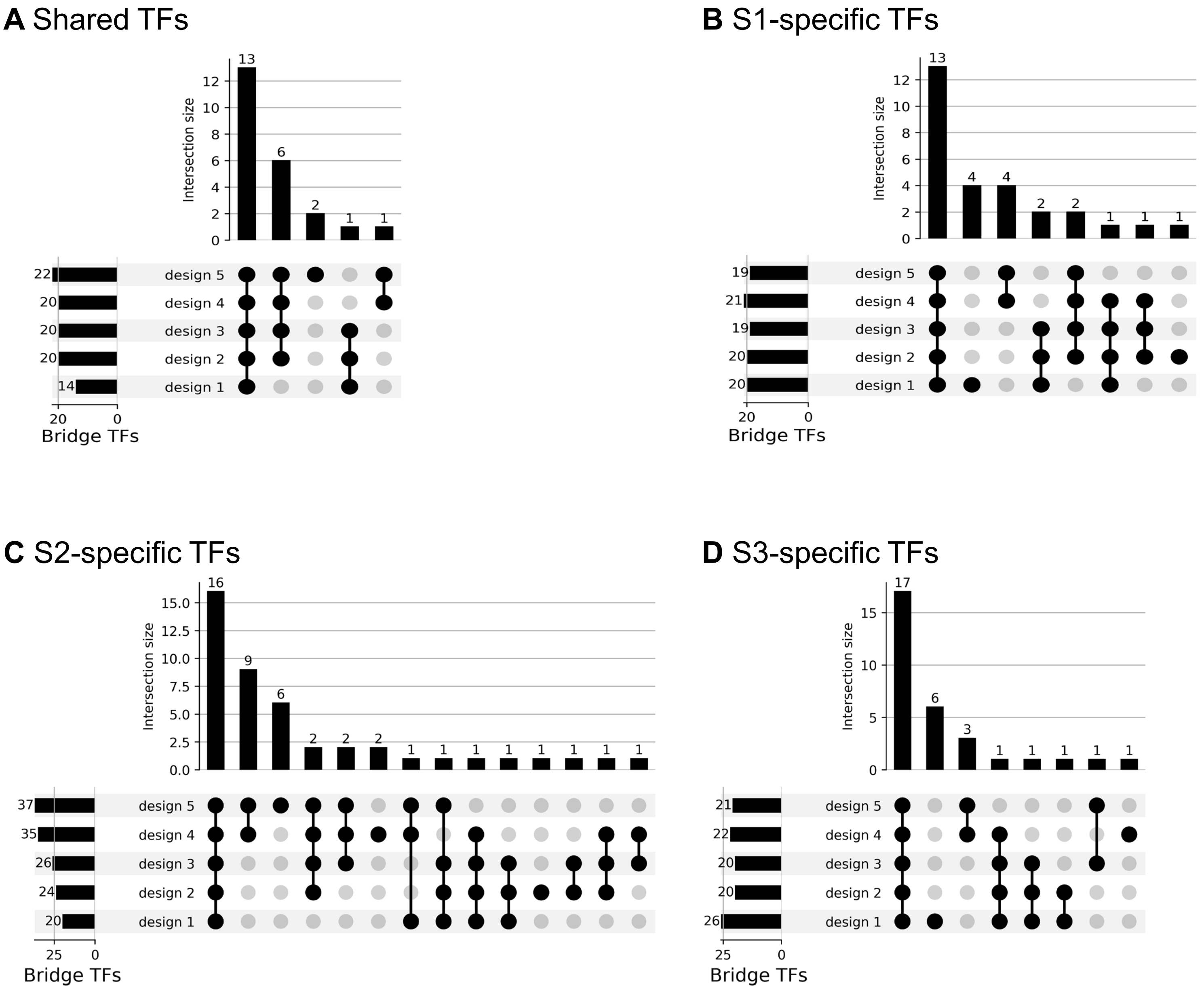

Supplementary Figure 7

Supplementary Figure 7

Five different design choices for the Steiner networks were tested and compared to analyze the robustness of design choices. Design 2 is the one described in the main text and used for all subsequent analyses. The only difference in Design 1 compared to 2 is that TFs from the next older network are not prized additionally, but every network is constructed independently. For Design 3-5, we also added protein-protein interaction edges between the target DE genes (red nodes in Fig. 2A). In Design 4, not only transcription factors, but also unprized nodes that were included in the next older network as well as their protein-protein interactions with the differentially expressed genes were added. Finally, in Design 5, these unprized nodes from the next older network were not only included, but also prized with a minimum prize.

A. Intersections of the shared bridge TFs (occurring in all 3 networks) over the 5 design choices.
B. Intersections of the bridge TFs only occurring in Steiner network S1 over the 5 design choices.
C. Intersections of the bridge TFs only occurring in Steiner network S2 over the 5 design choices.
D. Intersections of the bridge TFs only occurring in Steiner network S3 over the 5 design choices.

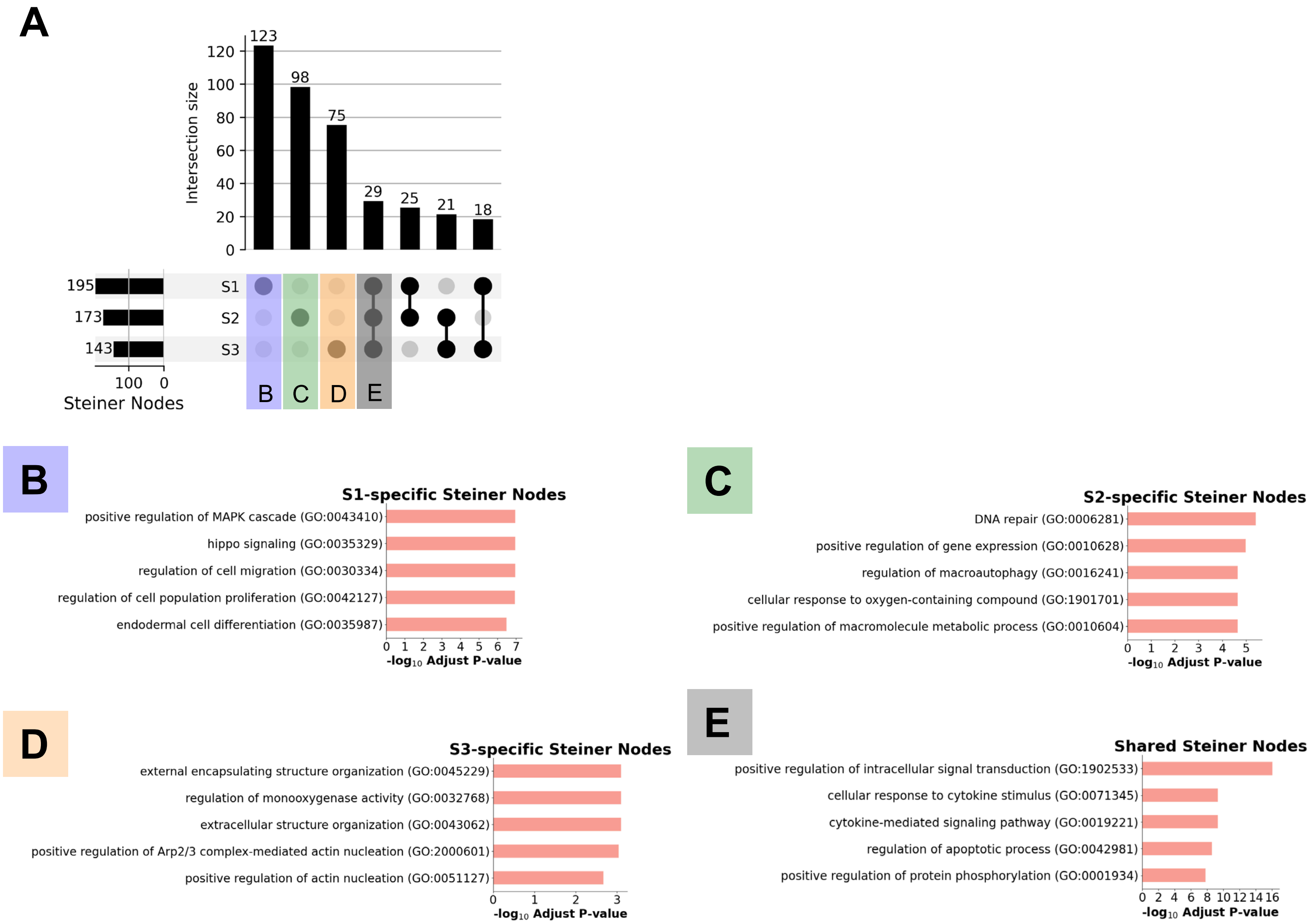

Supplementary Figure 8

Supplementary Figure 8

A. UpSet plot of Steiner nodes selected in the three Steiner networks (S1, S2, S3). The first four groups are marked using specific colors since the functions of these groups of genes are analyzed in B-E.
B. Gene Ontology Analysis of S1-specific Steiner nodes, using the gene set 'GO_Biological_Process_2021'. The top 5 pathways with lowest FDR-adjusted *p*-values ($p < 0.05$) are reported.
C. Gene Ontology Analysis of S2-specific Steiner nodes.
D. Gene Ontology Analysis of S3-specific Steiner nodes.
E. Gene Ontology Analysis of Steiner nodes shared between S1, S2, and S3.

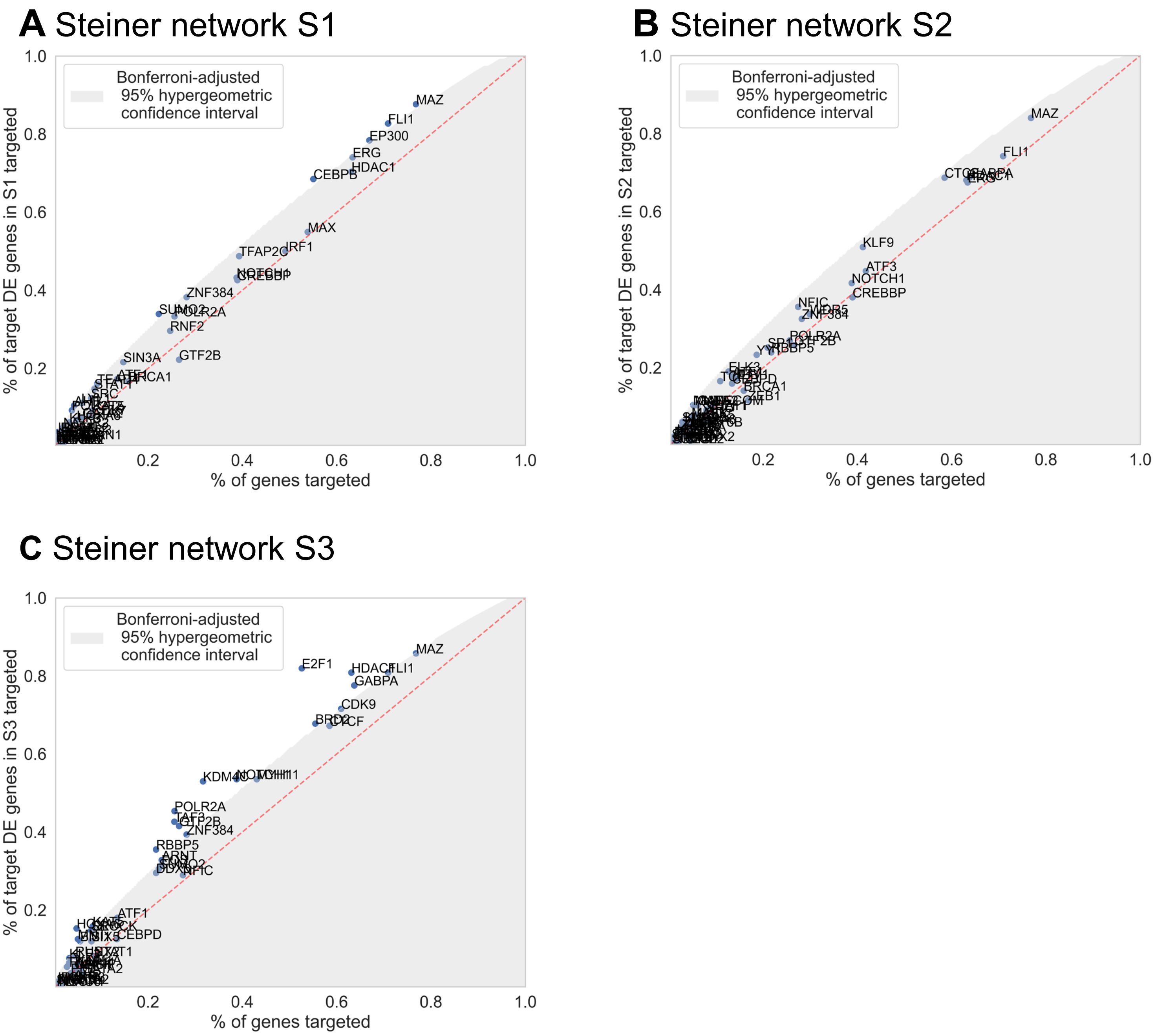

Supplementary Figure 9

Supplementary Figure 9

A. Scatterplot of bridge TFs in the Steiner network S1 based on the percentage of genes targeted overall in the genome (x-axis) and the percentage of target DE genes targeted (y-axis). The red line marks the identity function and the area shaded in grey corresponds to the Bonferroni-adjusted 95% hypergeometric confidence interval (see Methods).
B. Scatterplot of bridge TFs in the Steiner network S2.
C. Scatterplot of bridge TFs in the Steiner network S3.

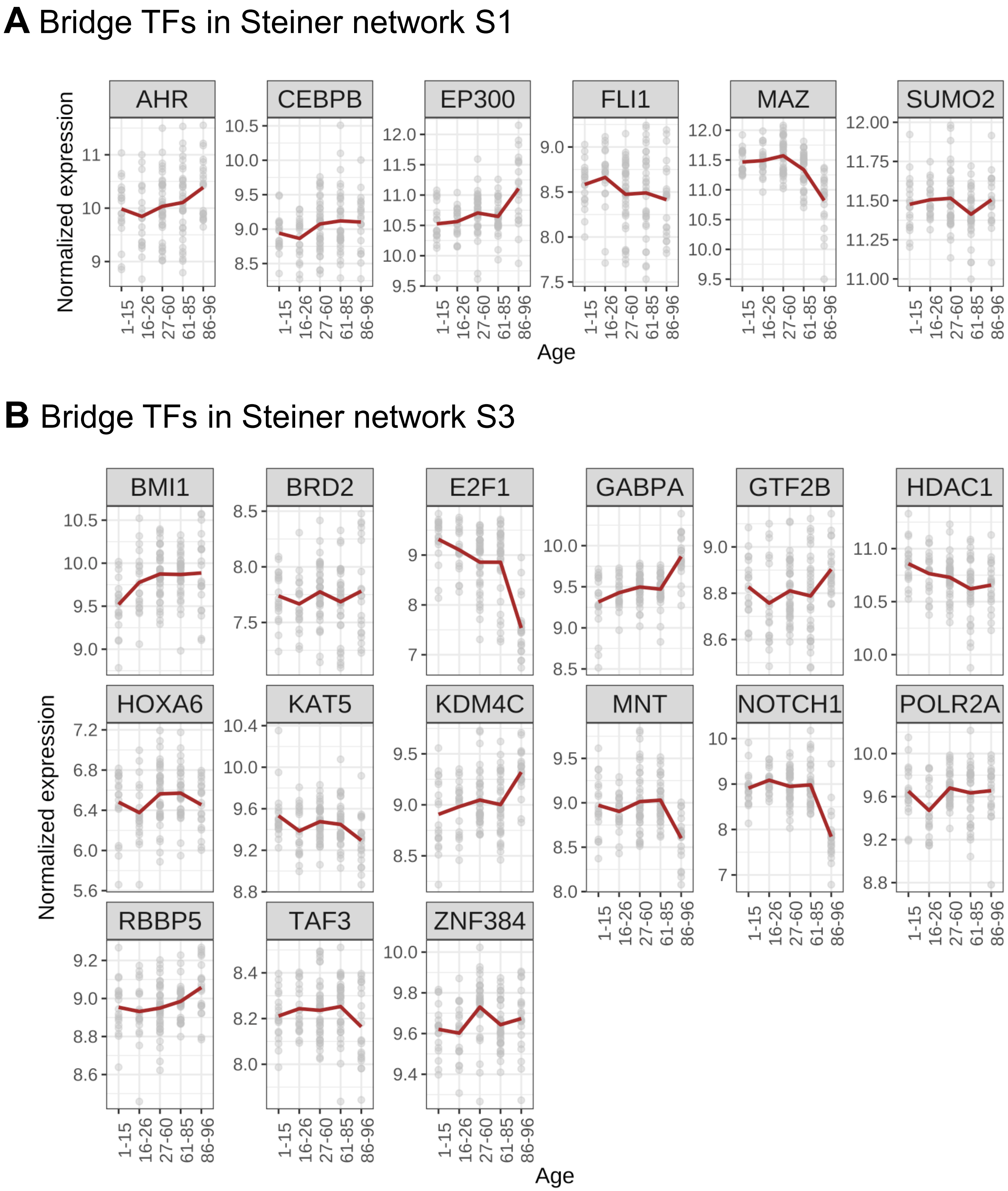

Supplementary Figure 10

Supplementary Figure 10

A. RNA-seq expression of significant bridge TFs in the Steiner network S1 (identified in the analysis in Supplementary Figure 9) over the 5 age groups. Each dot shows the variance-stabilized expression of one individual and the red line marks the mean expression in each age group.
B. RNA-seq expression of significant bridge TFs in the Steiner network S3 (identified in the analysis in Supplementary Figure 9) over the 5 age groups.

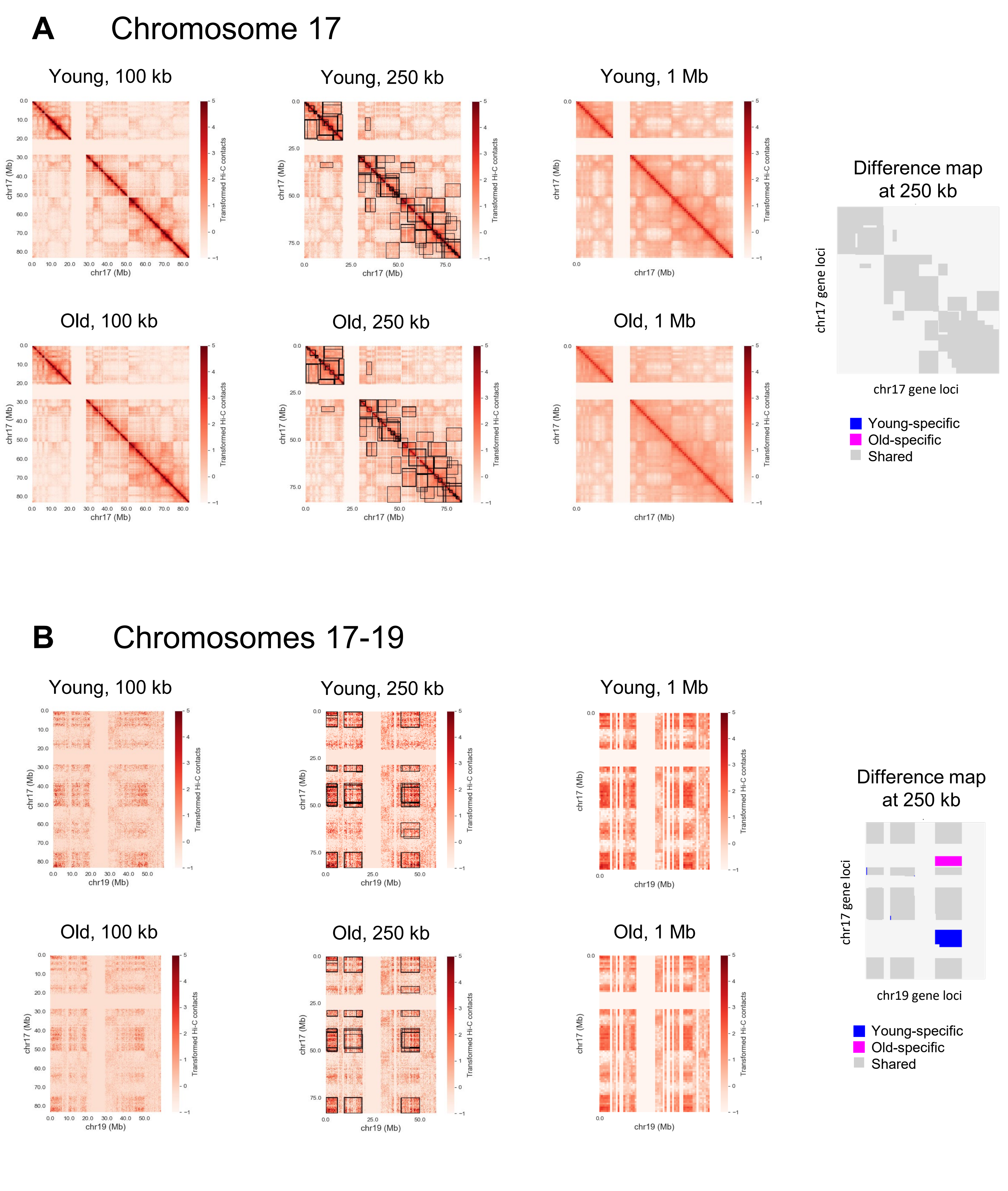

Supplementary Figure 11

Supplementary Figure 11

Selected Hi-C matrices for the young and old cell states at multiple resolutions (100 kb, 250 kb and 1 Mb). Hi-C contact values were preprocessed and centerdized (see Methods). For visualization, the mean contact values over the two replicates in the young and old replicates is shown for each resolution. Black boxes in the 250kb resolution indicate significant submatrices with high average values in both replicates that were selected by the Large Average Submatrix (LAS) algorithm. To compare these submatrices between the young and old state, intermingling difference maps for gene loci (excluding loci removed during preprocessing) were created. They show which of the LAS submatrices only occurred in young samples (blue), only in old samples (magenta) or in both samples (grey).

A. Intrachromosomal Hi-C matrices of chromosome 17.
B. Interchromosomal Hi-C matrices of chromosomes 17 and 19.

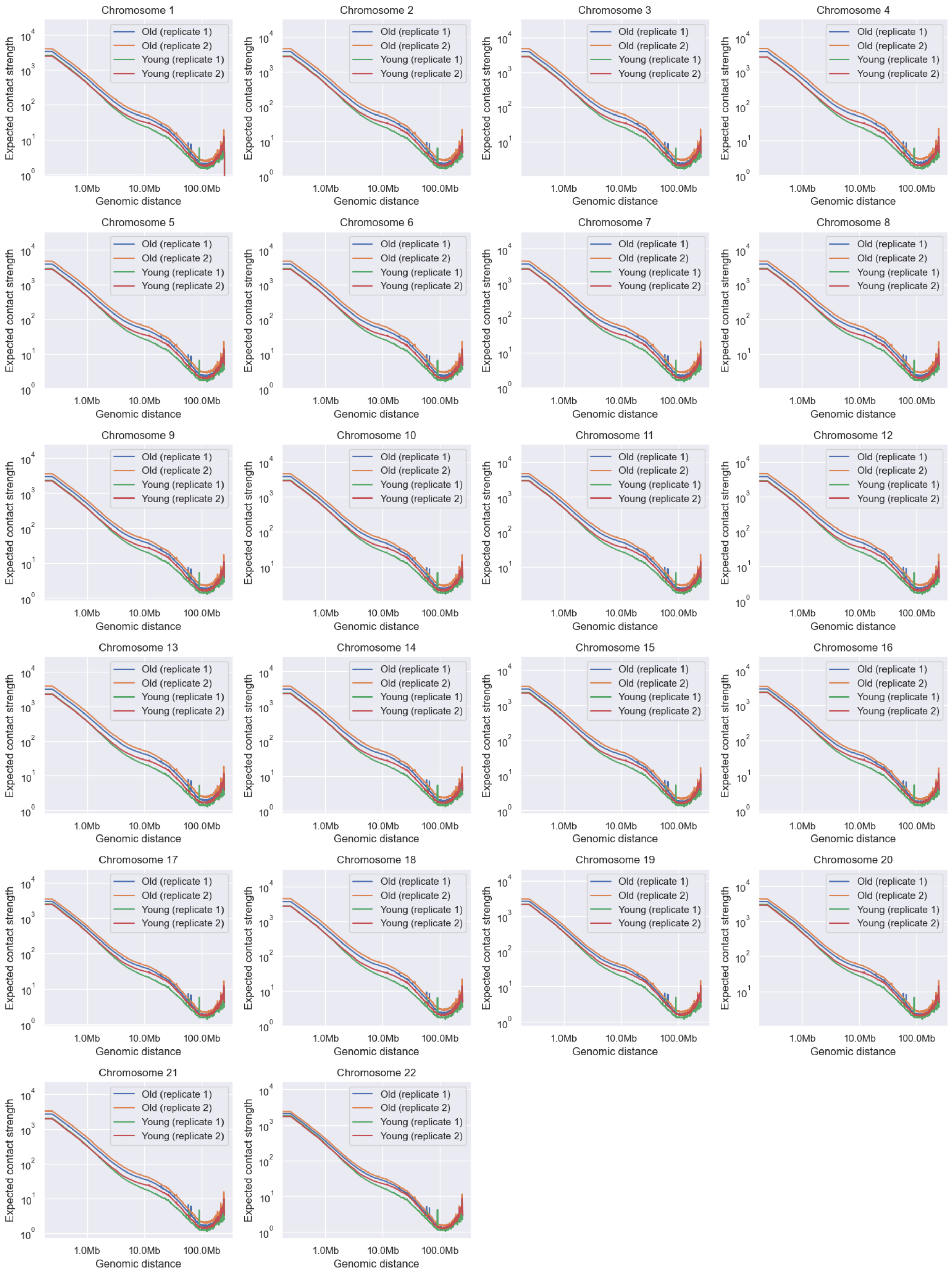

Supplementary Figure 12

Supplementary Figure 12

Distance decay plots for all chromosomes in the young and old Hi-C replicates displaying the expected Hi-C contact strength (y-axis) in pairs of genomic loci at a given genomic distance (x-axis). Intrachromosomal Hi-C maps were binned at the 250 kb resolution and balanced using the Knight-Ruiz algorithm.

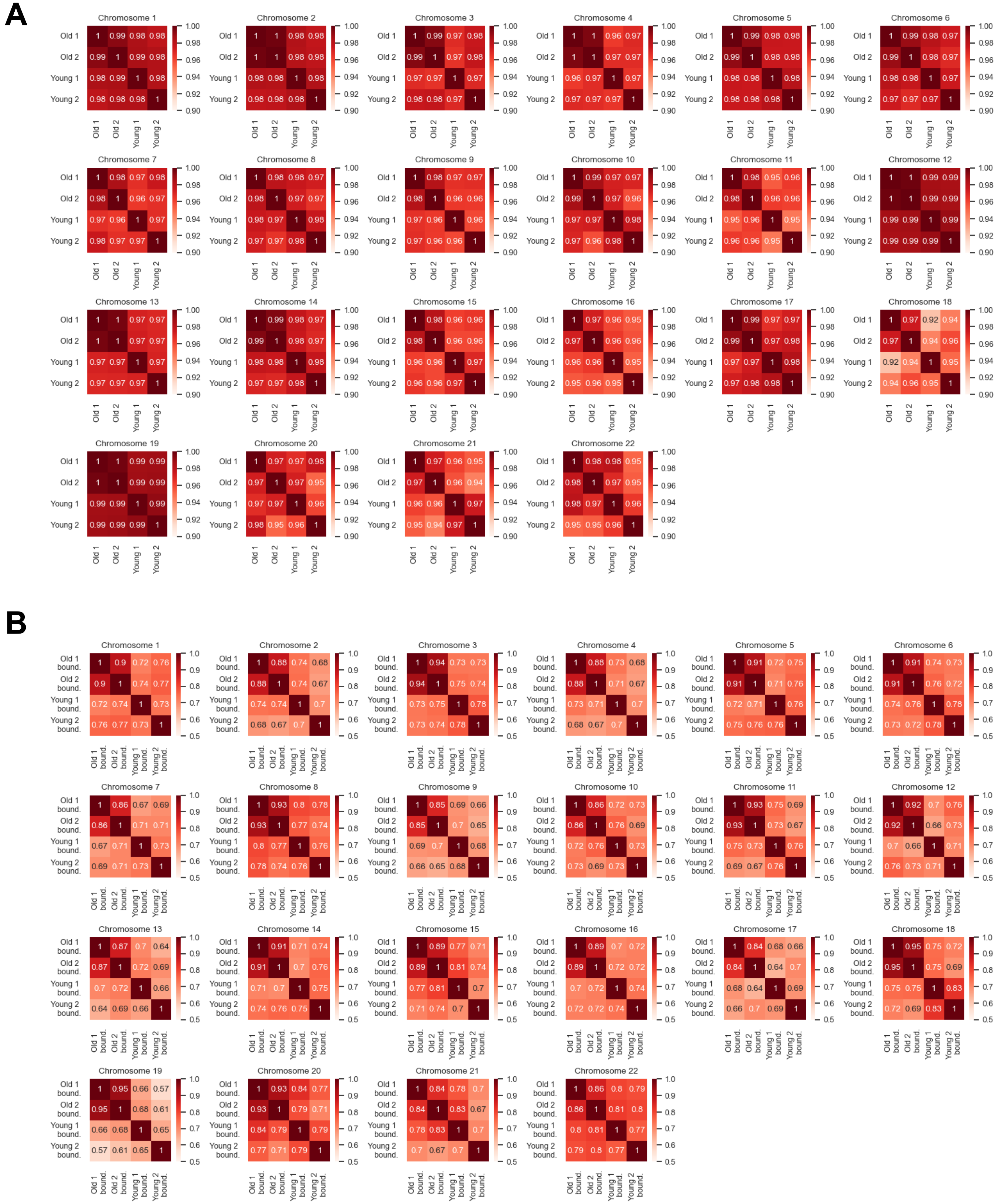

Supplementary Figure 13

Supplementary Figure 13

A. Spearman correlation matrices of insulation score profiles of both young Hi-C replicate maps and both old Hi-C replicate maps for all chromosomes. Insulation scores were computed from the balanced Hi-C matrices (using the Knight-Ruiz algorithm) binned at the 100 kb resolution, with a window size of 1 Mb.
B. Spearman correlation matrices of boundary score profiles of both young Hi-C replicate maps and both old Hi-C replicate maps for all chromosomes. Boundary score profiles were obtained by calling all minima from the insulation score profiles in A.

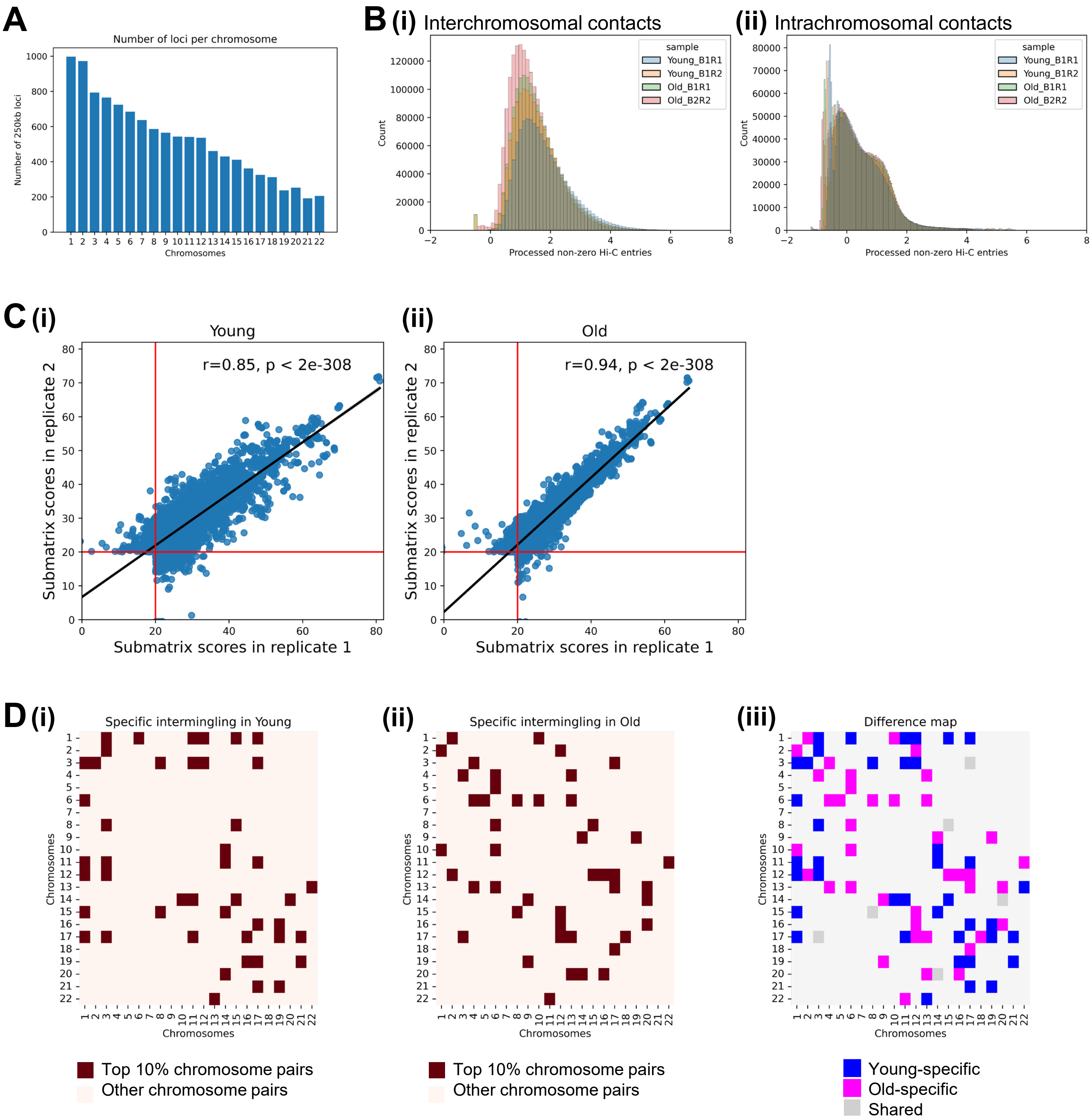

Supplementary Figure 14

Supplementary Figure 14

A. Distribution of the number of 250 kb loci per chromosome (excluding chromosomes X, Y, and M).
B. Distribution of z-scored non-zero interchromosomal contacts (B(i)) and intrachromosomal contacts (B(ii)) over all chromosome pairs in the two young and two old Hi-C replicates. Note that the histograms are not centered at 0 because z-scores are calculated over all Hi-C entries, but only the z-scores corresponding to non-zero values are shown here.
C. Comparison of interchromosomal LAS scores between Hi-C replicates of the same cell line. Since two replicates of Hi-C data from a young individual (10-year-old) and from an old individual (75-year-old) were generated, the LAS score of each submatrix in replicate 1 (x-axis) versus its score in replicate 2 (y-axis) for the young individual (C(i)) and for the old individual (C(ii)) are shown. The red lines mark the threshold of 20 that was used in the LAS algorithm (see Methods). Only submatrices that were above the threshold in both replicates were kept for downstream analyses. The correlation coefficient r, as well as the *p*-value of the correlation test are shown.
D. Distribution of intermingling interactions per chromosome pair. For each chromosome pair, the number of loci pairs that were part of a submatrix only in young (D(i)) or only in old (D(ii)) was calculated and the top 10% chromosome pairs with most cell-state-specific intermingling are marked in dark red. D(iii) visualizes a comparison of the chromosome pairs with most young-specific and old-specific intermingling, Chromosome pairs that were only part of the top 10% in young are shown in blue, chromosome pairs only part of the top 10% in old in magenta and chromosome pairs with many cell-type-specific intermingling interactions in young and old in grey.

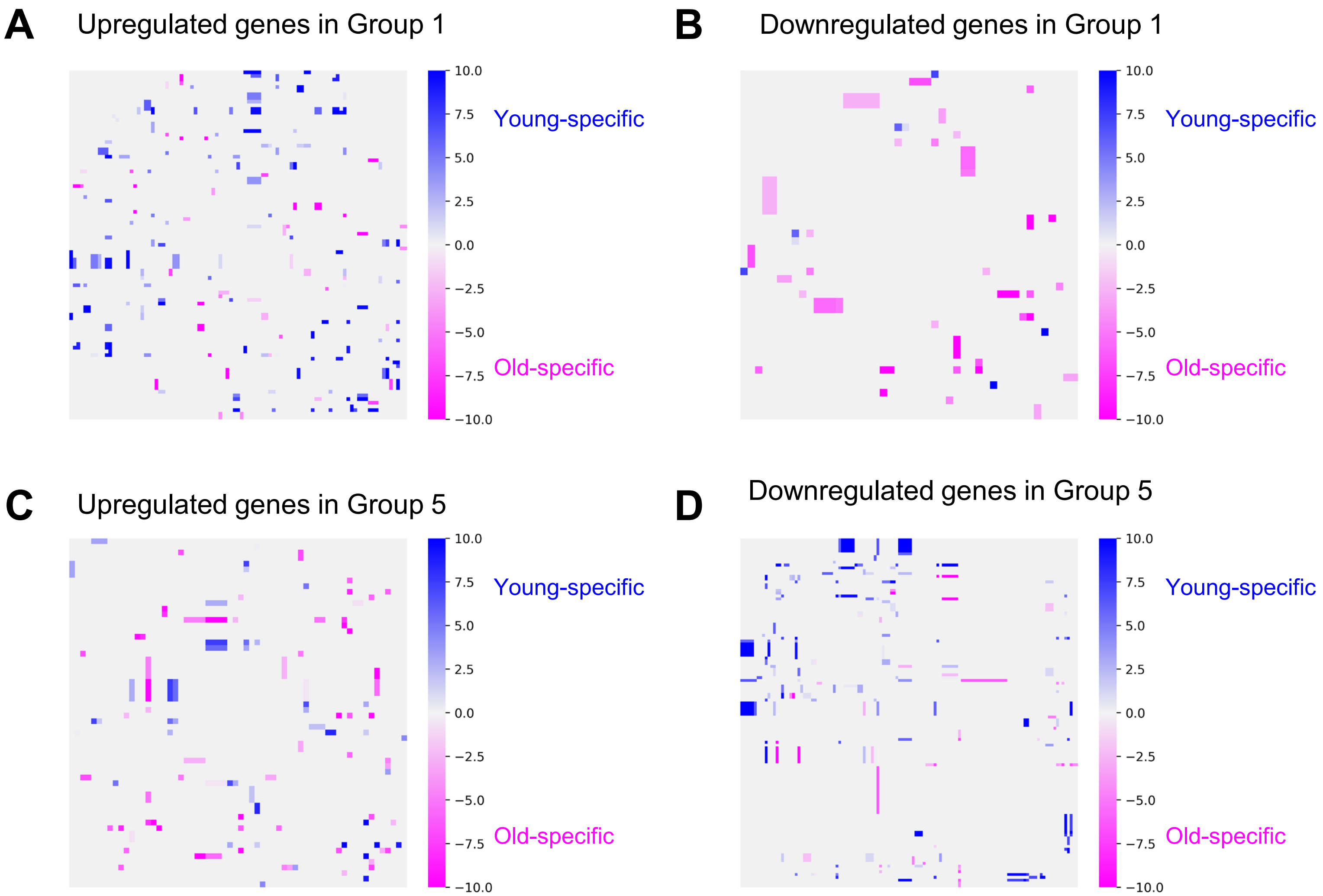

Supplementary Figure 15

Supplementary Figure 15

Intermingling difference maps for selected age-associated DE genes. Young-specific LAS submatrices are colored in shades of blue and old-specific LAS submatrices are shown in shades of magenta based on the difference of their respective LAS scores (see Methods) between the young Hi-C replicates and the old Hi-C replicates. The selected genes are sorted in ascending genomic order.

A. Upregulated genes in Group 1 with specific intermingling (upper left quadrant of Figure 4A(ii)).
B. Downregulated genes in Group 1 with specific intermingling (upper left quadrant of Figure 4B(ii)).
C. Upregulated genes in Group 5 with specific intermingling (upper left quadrant of Figure 4C(ii)).
D. Downregulated genes in Group 5 with specific intermingling (upper left quadrant of Figure 4D(ii)).

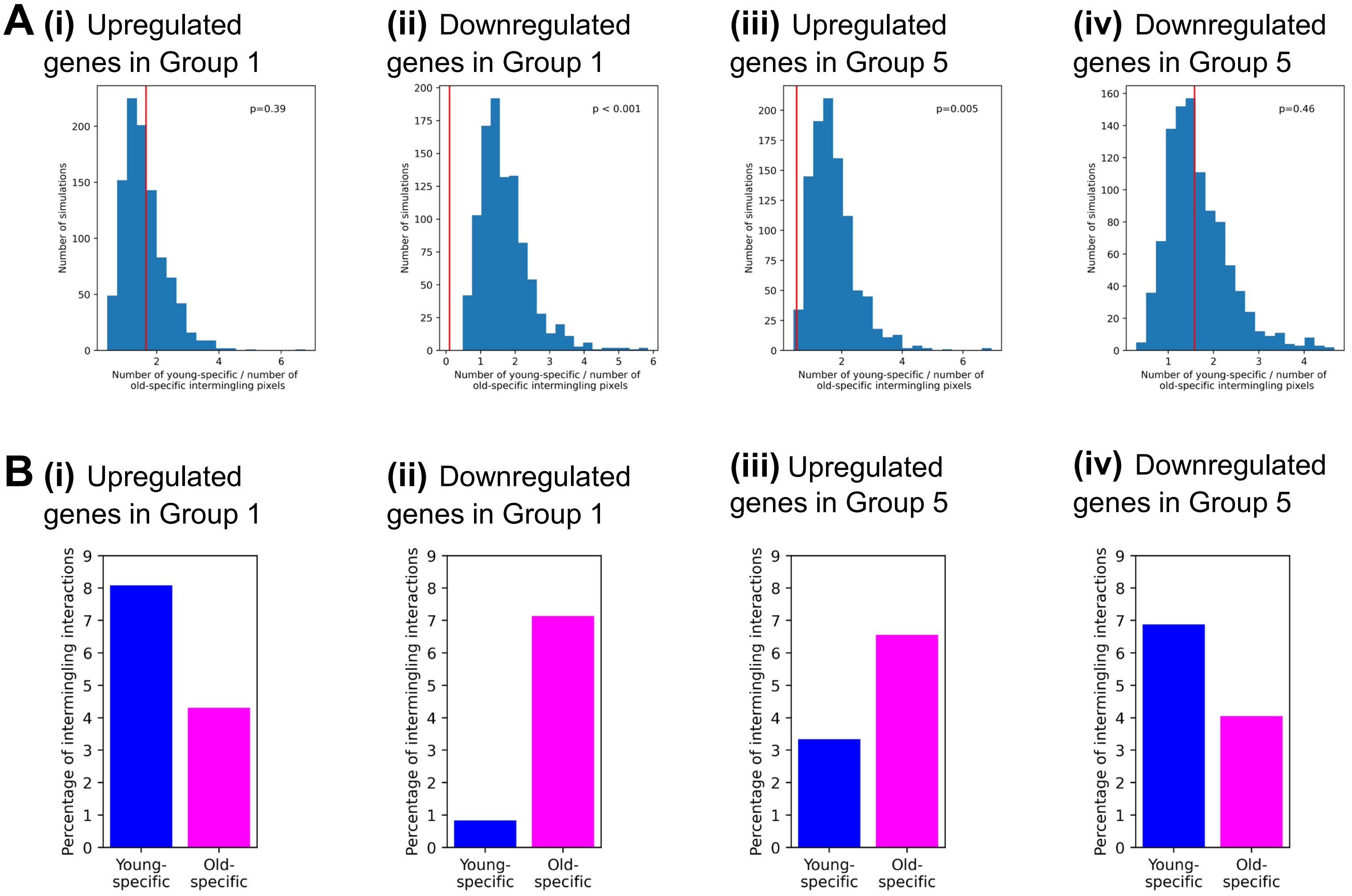

Supplementary Figure 16

Supplementary Figure 16

A. Histograms for the intermingling ratio in 1000 simulations creating intermingling difference maps for 170 random genes (see Methods) used as null model. The intermingling ratio of an intermingling difference map is defined as the number of young-specific intermingling entries divided by the number of old-specific intermingling entries. These distributions (blue) were compared to the ratios for the intermingling maps in Figure 4 (red vertical line): DE genes upregulated in Group 1 (A(i)), DE genes downregulated in Group 1 (A(ii)), DE genes upregulated in Group 5 (A(iii)), and DE genes downregulated in Group 5 (A(iv)).
B. Bar plots quantifying the percentage of young- and old-specific intermingling entries out of all intermingling interactions in the intermingling difference maps for the upregulated DE genes in Group 1 (B(i)), downregulated DE genes in Group 1 (B(ii)), upregulated DE genes in Group 5 (B(iii)) and downregulated DE genes in Group 5 (B(iv)).

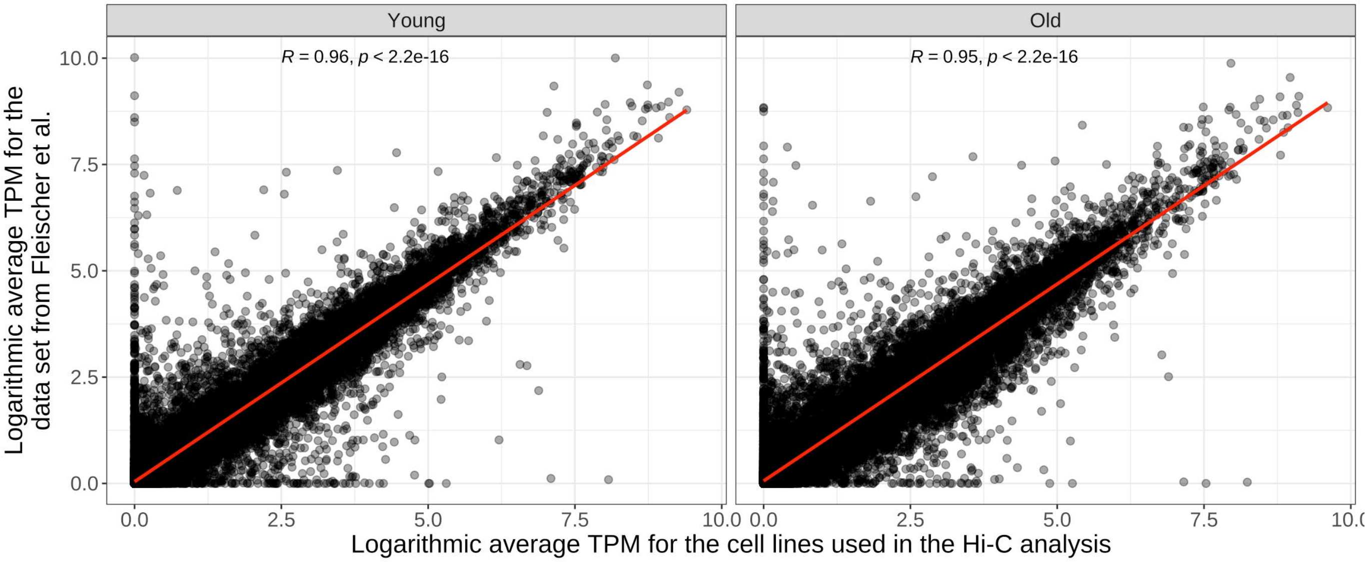

Supplementary Figure 17

Supplementary Figure 17

Comparison of the transcriptomics data in young (left panel) and old fibroblasts (right panel) from the cell lines used in the Hi-C analysis (x-axis) and the data set from Fleischer et al. which was used for the differential gene expression analysis in our work (y-axis). For the Hi-C cell lines, we used the average TPM over two replicates (young: GM09503 and old: GM08401) and for the data from Fleischer et al., we used the average TPM over Group 1 (young) and Group 5 (old). The average expression of each gene (black dots) is visualized as the log(TPM +1)) transformation. The fitted regression line is shown in red, and the correlation coefficient R and the *p*-value of the correlation test were added to the two panels.

A

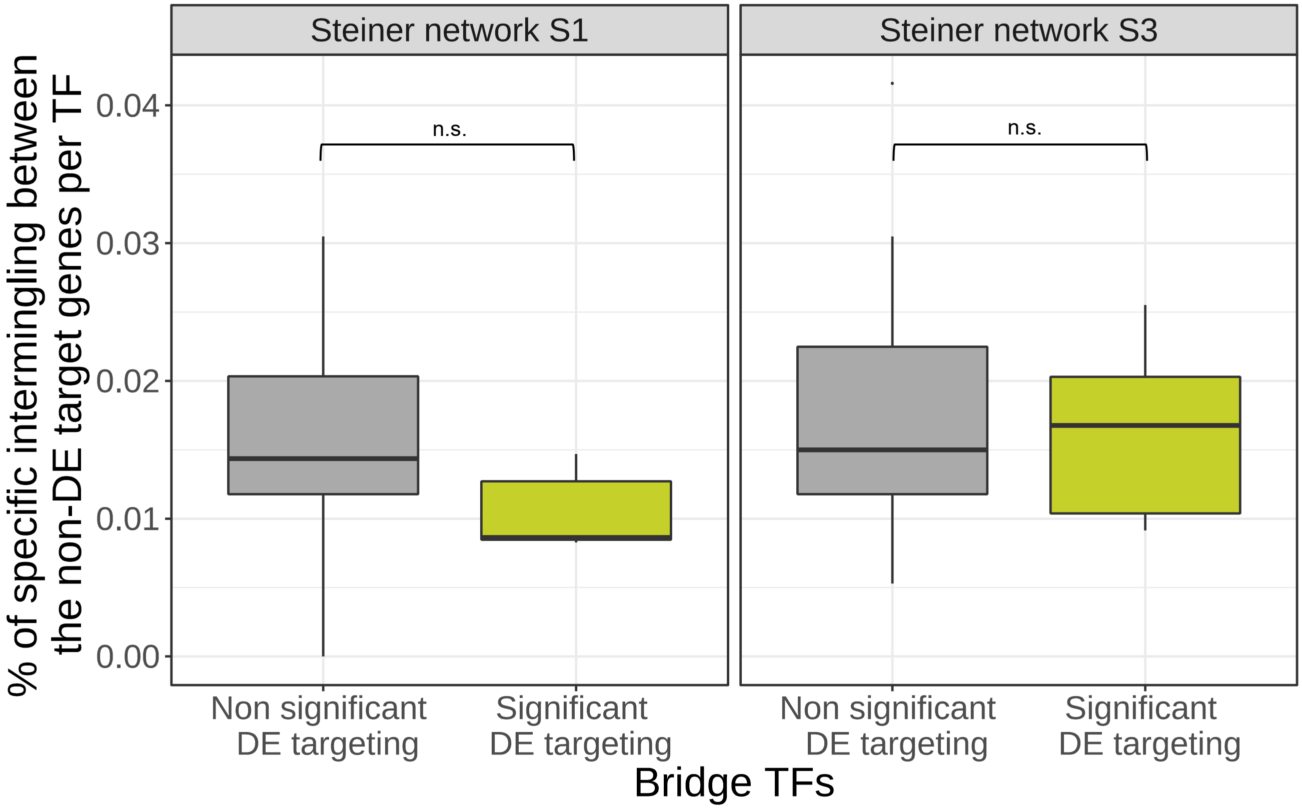

B

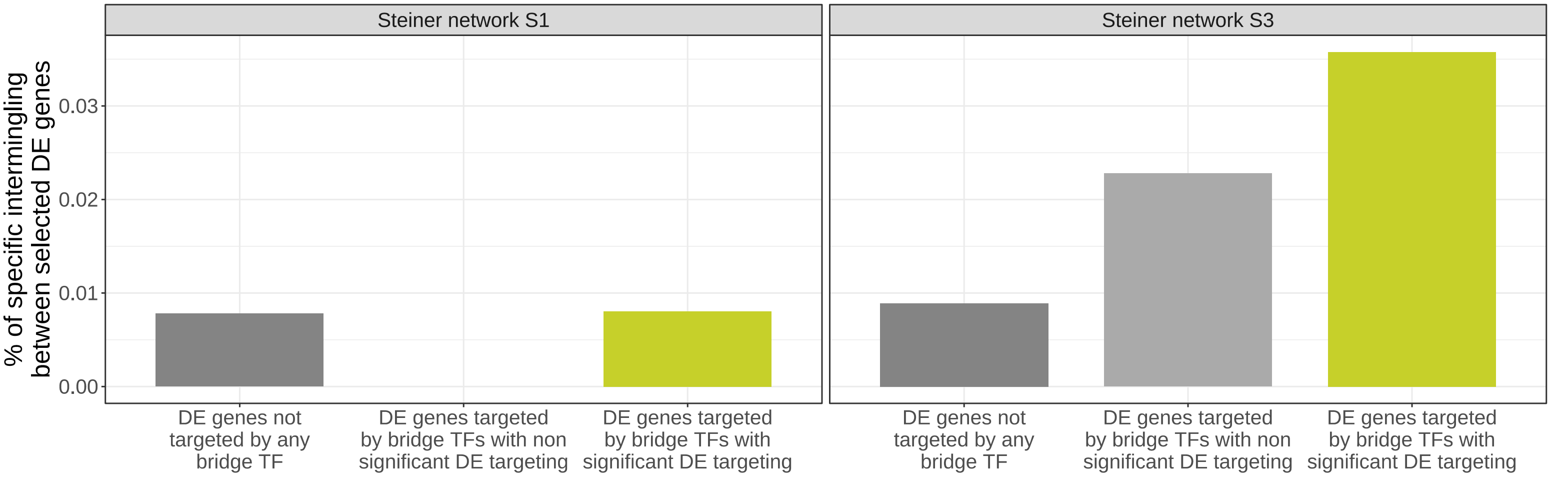

Supplementary Figure 18

Supplementary Figure 18

A. Distribution of the proportion of age-specific intermingling among non-DE genes targeted by significant bridge TFs (green box) and non-significant bridge TFs (grey box) from Steiner networks S1 (left) and S3 (right). Significant bridge TFs correspond to the TFs in Fig. 2F.
B. Proportion of age-specific intermingling among DE genes targeted by significant bridge TFs (green bar), DE genes targeted by non-significant bridge TFs (light grey bar), and DE genes that are not targeted by any bridge TF (dark grey bar, 22 genes in S1 and 18 genes in S3) from Steiner networks S1 (left) and S3 (right). The green and light grey bars correspond to the median value of the similarly colored boxplots in Fig. 5B.

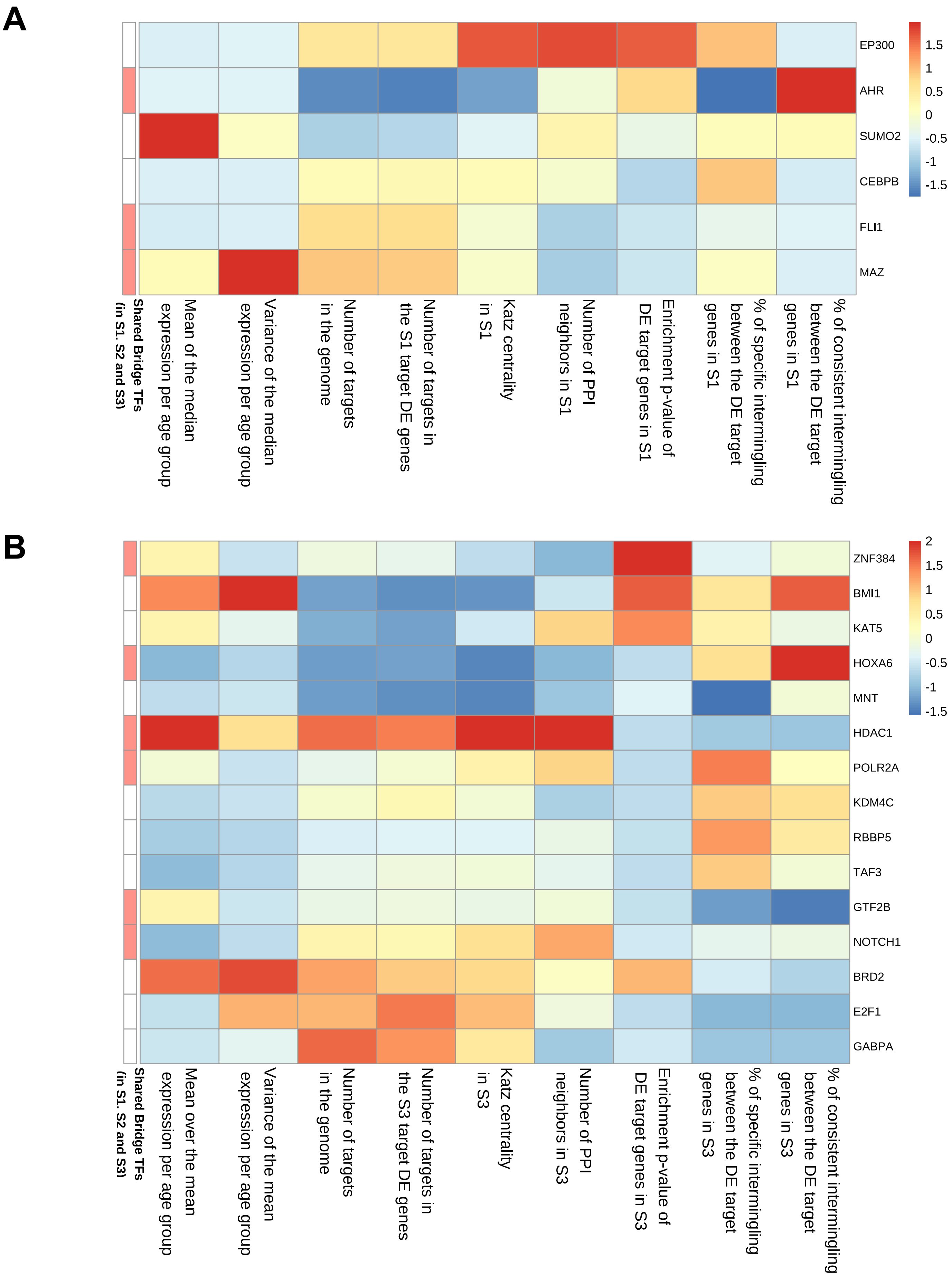

Supplementary Figure 19

Supplementary Figure 19

A. Heatmap with hierarchical clustering of the significant bridge TFs (y-axis) in the Steiner network S1 for multiple features (x-axis) using Euclidean metric and complete linkage. The selected features are: (i) mean RNA-seq expression of the TF over the 5 age groups, (ii) variance of its RNA-seq expression over the five age groups, (iii) number of target genes in the genome, (iv) number of target DE genes targeted by the TF, (v) Katz centrality of a TF in the Steiner network, (vi) number of protein-protein interactions of the TF in the Steiner network, (vii) *p*-value for the enrichment in DE gene targeting from the hypergeometric test of Fig. 2F, (viii) proportion of intermingling interactions between the target DE genes targeted by the TF that were only part of an LAS submatrix in young or old Hi-C data, but not in both, and (ix) proportion of intermingling interactions between the target DE genes targeted by a TF that were part of an LAS submatrix in both young and old Hi-C data. Values were z-scored for each feature and clipped to the [-2, 2] interval. Only features (iv) – (viii) were used for clustering the TFs. The annotation bar on the left side of the heatmap marks bridge TFs included in all three Steiner networks (S1, S2 and S3) in red.

B. Heatmap with hierarchical clustering of the significant bridge TFs (y-axis) in the Steiner network S3.

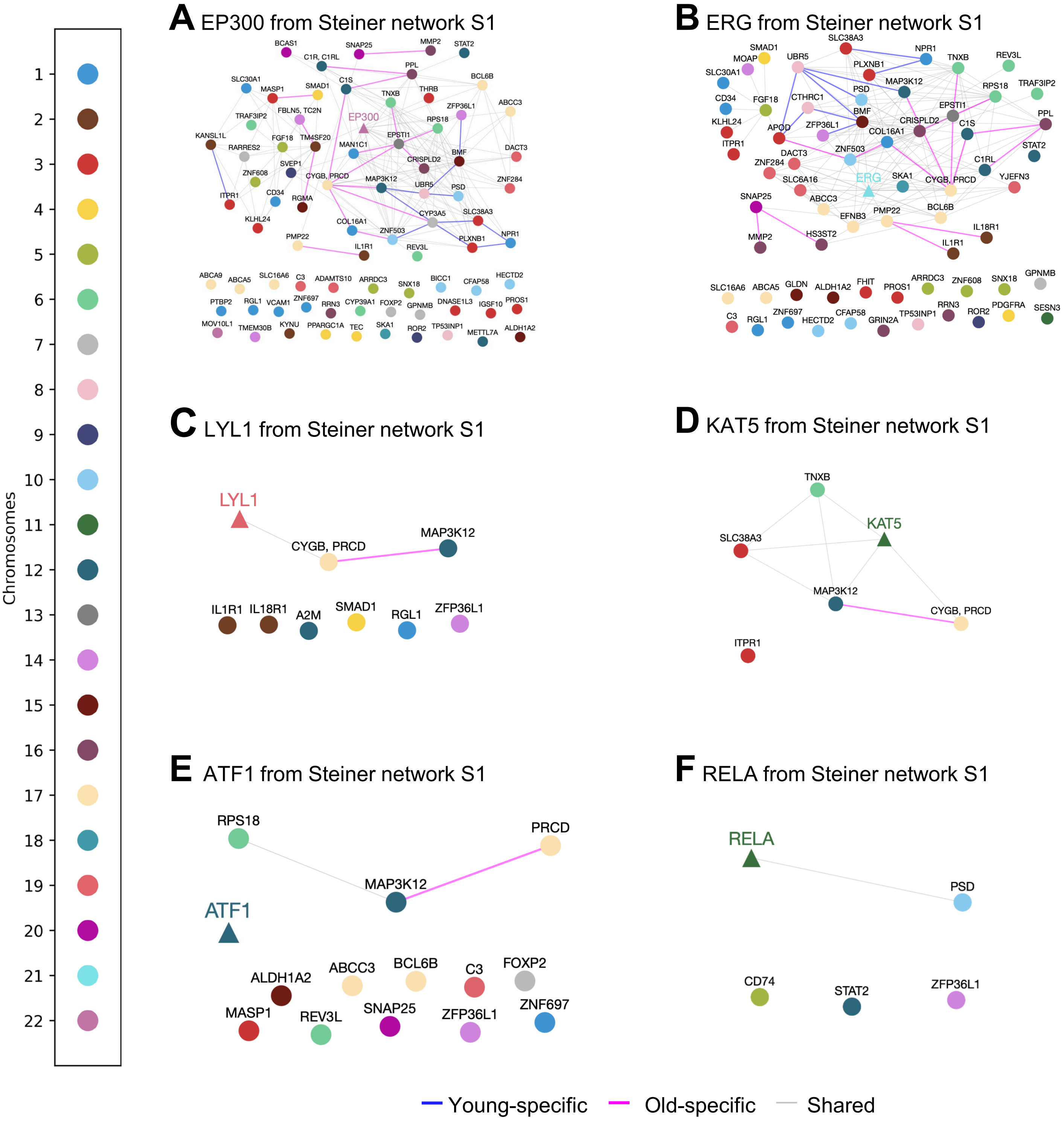

Supplementary Figure 20

Supplementary Figure 20

Intermingling network for various bridge TFs with their upregulated DE targets in Steiner network S1.

A. EP300 (low *p*-value for targeting DE genes and a high percentage of cell-state-specific intermingling, see Fig. 5C1)
B. ERG (low *p*-value for targeting DE genes and a high percentage of cell-state-specific intermingling, see Fig. 5C1)
C. LYL1 (among the highest percentage of cell-state-specific intermingling in the heatmap of Fig. 5C1)
D. KAT5 (among the highest percentage of cell-state-specific intermingling in the heatmap of Fig. 5C1)
E. ATF1 (negative example that is not enriched in DE targeting)
F. RELA (negative example that is not enriched in DE targeting)

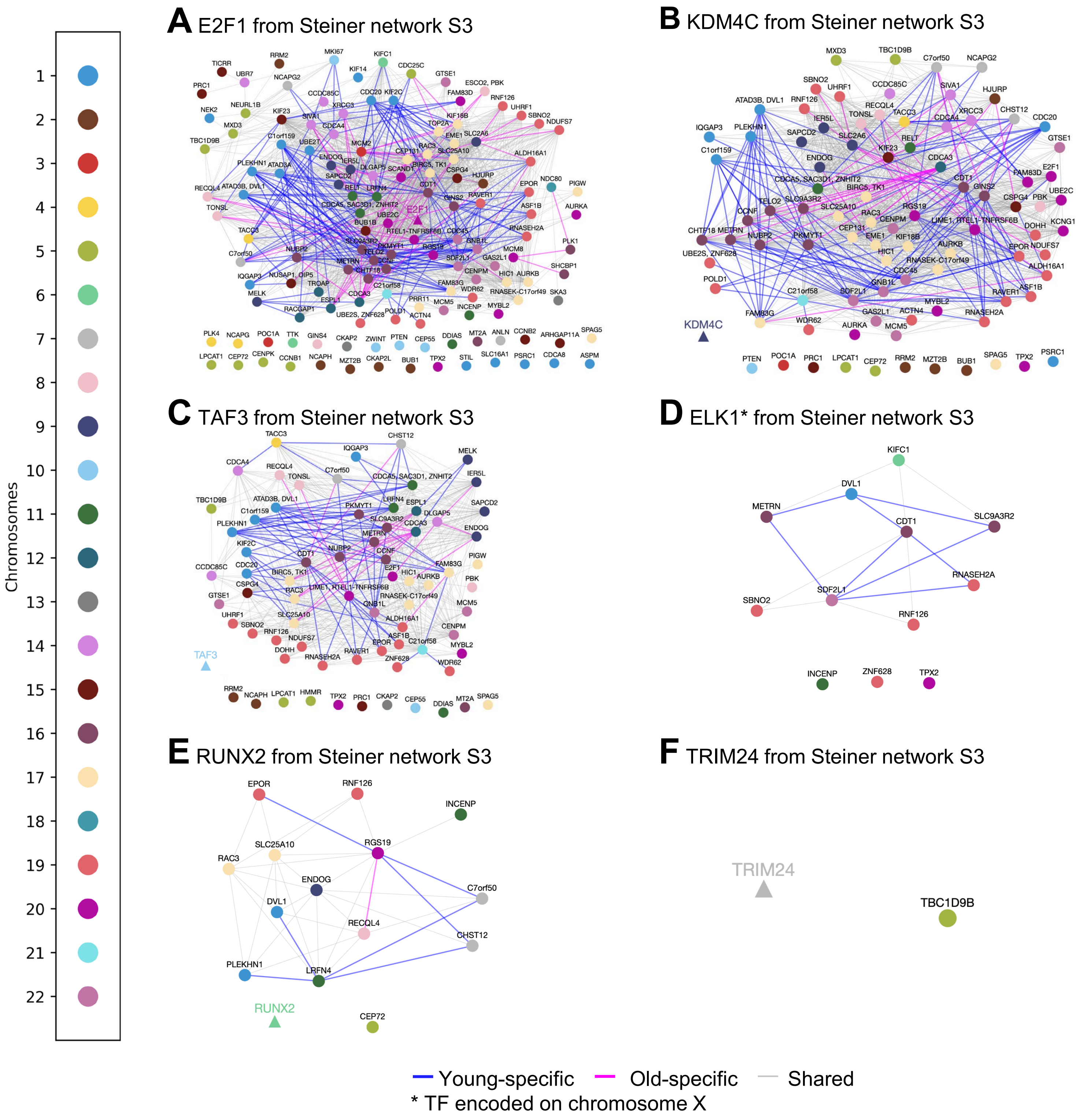

Supplementary Figure 21

Supplementary Figure 21

Intermingling networks for various bridge TFs with their downregulated DE targets in Steiner network S3.

A. E2F1 (among the lowest *p*-values for DE gene targeting and has a high percentage of cell-state-specific intermingling)
B. KDM4C (among the lowest *p*-values for DE gene targeting and has a high percentage of cell-state-specific intermingling)
C. TAF3 (among the lowest *p*-values for DE gene targeting and has a high percentage of cell-state-specific intermingling)
D. ELK1 (this TF is encoded on chromosome X and is therefore not included in its intermingling network)
E. RUNX2 (highest percentage of intermingling between its DE targets in Fig. 5C2)
F. TRIM24 (negative example with no DE enrichment and no intermingling changes)
